# Differential responses of canonical nitrifiers and comammox *Nitrospira* to long-term fertilization in an Alfisol of Northeast China

**DOI:** 10.3389/fmicb.2023.1095937

**Published:** 2023-02-01

**Authors:** Yanan Wang, Xibai Zeng, Qiang Ma, Yang Zhang, Wantai Yu, Zhong Zheng, Nan Zhang, Liyang Xu

**Affiliations:** ^1^Institute of Environment and Sustainable Development in Agriculture, Chinese Academy of Agricultural Sciences, Beijing, China; ^2^Insitute of Applied Ecology, Chinese Academy of Sciences, Shenyang, China

**Keywords:** comammox *Nitrospira*, recycled manure, soil layer, phylogenetic analysis, structural equation modeling analysis, niche differentiation

## Abstract

The newly identified complete ammonia oxidizer (comammox) that converts ammonia directly into nitrate has redefined the long-held paradigm of two-step nitrification mediated by two distinct groups of nitrifiers. However, exploration of the niche differentiation of canonical nitrifiers and comammox *Nitrospira* and their ecological importance in agroecosystems is still limited. Here, we adopted quantitative PCR (qPCR) and Illumina MiSeq sequencing to investigate the effects of five long-term fertilization regimes in the variations of ammonia-oxidizing bacteria (AOB), ammonia-oxidizing archaea (AOA), nitrite-oxidizing bacteria (NOB), and comammox *Nitrospira* abundances and comammox community composition in two soil layers (0–20 cm, topsoil; 20–40 cm, subsoil) in an Alfisol in Northeast China. The fertilization treatments included no fertilizer (CK); chemical nitrogen (N) fertilizer; chemical N; phosphorus (P) and potassium (K) fertilizers (NPK); recycled organic manure (M) and chemical N, P, K plus recycled manure (MNPK). Compared with CK, manure and/or chemical fertilizer significantly increased the AOB *amoA* gene abundance. Long-term recycled manure increased soil organic matter (SOM) contents and maintained the soil pH, but decreased the NH_4_^+^-N concentrations, which markedly promoted the *nxrA* and *nxrB* gene abundances of NOB and the *amoA* gene abundances of comammox *Nitrospira* clade A and AOA. Although the comammox *Nitrospira* clade B abundance tended to decrease after fertilization, the structural equation modeling analysis showed that comammox clade B had direct positive impacts on soil potential ammonia oxidation (PAO; λ = 0.59, *p* < 0.001). The long-term fertilization regime altered the community composition of comammox *Nitrospira*. Additionally, comammox *Nitrospira* clades A and B had individual response patterns to the soil layer. The relative abundance of clade A was predominant in the topsoil in the N (86.5%) and MNPK (76.4%) treatments, while clade B appeared to be dominant in the subsoil (from 78.7 to 88.1%) with lower ammonium contents, implying niche separation between these clades. Soil pH, NH_4_^+^-N and SOM content were crucial factors shaping the soil nitrifying microbial abundances and the comammox *Nitrospira* community. Together, these findings expand the current understanding of the niche specialization and the important role of comammox *Nitrospira* in terrestrial ecosystems.

## 1. Introduction

In nature, microbial-mediated nitrification processes play a crucial role in the transformation of nitrogen (N), resulting in nitrogen flux from terrestrial ecosystems and water eutrophication resulting from nitrate leaching (Hu et al., 2014; Kuypers et al., 2018). The biological oxidation of ammonia (NH_3_) to nitrate (NO_3_^−^) *via* nitrite (NO_2_^−^), known as classical two-step nitrification, is an important process in the global nitrogen cycle. The first step of nitrification, namely ammonia oxidation, is catalyzed by ammonia-oxidizing bacteria (AOB) and ammonia-oxidizing archaea (AOA; Könneke et al., 2005). The second step, namely, nitrite oxidation, is performed by nitrite-oxidizing bacteria (NOB). In 2015, the complete ammonia oxidation (comammox) process was reported to oxidize ammonia to nitrate in a single cell, which fundamentally challenged the long-held perspective of the nitrification process (Daims et al., 2015; van Kessel et al., 2015).

To date, all of the comammox *Nitrospira* identified belong to *Nitrospira* sublineage II, harboring an entire set of nitrifying genes including ammonia monooxygenase (AMO), hydroxylamine dehydrogenase (HAO) and nitrite oxidoreductase (NXR; van Kessel et al., 2015; Poghosyan et al., 2019). Metagenomic evidence suggests that the comammox *Nitrospira amoA* gene is phylogenetically different from that of canonical ammonia-oxidizing microorganisms (AOA and AOB). Subsequently, based on the phylogeny of the *amoA* gene, comammox *Nitrospira* has been classified into two separate clades including clade A and clade B (Daims et al., 2015), and the average nucleotide identity values between the genomes of clade A and clade B are in the range of 70 to 72% (Poghosyan et al., 2019). Moreover, clade A can be split into clades A1, A2, and A3 (Xia et al., 2018; Li et al., 2020).

Since the discovery of comammox *Nitrospira* as a novel ammonia-oxidizing member, the significant importance of different ammonia oxidizers to nitrification has become more complicated and diversified under various soil conditions. Theoretical calculations indicated that comammox *Nitrospira* prefer to exist in a low-substrate-concentration environment, and kinetic results confirmed that comammox *Nitrospira* had high affinity for the substrates ammonia and oxygen, demonstrating competitive advantages over canonical nitrifiers in oligotrophic habitats (Kits et al., 2017). However, Li et al. (2019) demonstrated that the addition of (NH_4_)_2_SO_4_ significantly stimulated the *amoA* gene abundance of *Nitrospira* clade A in a pasture soil based on a microcosm experiment. Gonzalez et al. (2016) and Palomo et al. (2016) analyzed comammox *Nitrospira* bacterial genome data and predicted that comammox *Nitrospira* might have versatile metabolization capability. Numerous metagenomic findings further verified that comammox *Nitrospira* could competently utilize urea, formate, hydrogen, cyanate, methane, and agmatine as well as degrade organic carbon, highlighting their metabolic flexibility beyond the forms of nitrogen (Palomo et al., 2018; Koch et al., 2019; Vijayan et al., 2020; Yang et al., 2020). In addition to ammonia oxidation, comammox *Nitrospira* are also capable of oxidizing nitrite, because nitrite oxidoreductase is conserved and highly similar in all *Nitrospira* genomes (Palomo et al., 2018). It is speculated that the nitrite affinity of *Nitrospira inopinata* is approximately 50 times higher than that of canonical *Nitrospira* (Kits et al., 2017). The unique feature of comammox *Nitrospira* genome seems to be the apparent absence of genes for assimilatory nitrite reduction, resulting in the loss of the potential to use external nitrite nitrogen sources (Palomo et al., 2018). Overall, it is essential to bridge our knowledge gap concerning the ecological characteristics of comammox *Nitrospira* and re-evaluate the niche specialization of different soil nitrifying groups in terrestrial ecosystems.

Chemical and manure fertilizers have been extensively applied in agricultural soils to maintain soil fertility and improve crop productivity. Long-term experiments with different fertilization regimes are crucial for examining the variations in soil properties and nutrient supply capacity, and they can be used to explore the mechanisms of numerous ecological processes in detail. Many previous studies pointed out that long-term application of different fertilization regimes can significantly impact the abundances and community compositions of nitrogen-related microorganisms, influencing the contributions and predominance of diverse nitrifier and denitrifier communities involved in nitrogen cycling in soils (Zhang et al., 2012; Gao et al., 2018; Hou et al., 2018). Wang et al. (2019) demonstrated that long-term N and phosphorus (P) fertilizer inputs significantly enhanced AOB *amoA* gene abundances, while AOA *amoA* gene abundances were not as sensitive as those of AOB. AOA and AOB exhibited higher *amoA* gene transcriptional abundances than comammox *Nitrospira* under long-term chemical nitrogen fertilizer treatment (Wang et al., 2021). In comparison, long-term manure fertilization dramatically increased comammox *Nitrospira* abundances and influenced their community compositions (Li et al., 2020). Manure fertilizer was also shown to improve the composition and diversity of *Nitrospira* and *Nitrobacter* NOB in an acidic red soil (Han et al., 2018). Nevertheless, to our knowledge, although various studies have been conducted to illustrate the changes in nitrifying microbial communities in response to manure application, most of these manure fertilizers were commercial manure or were from external sources, not from within the farmland ecosystem. In fact, manure fertilizers from different sources vary greatly in their nutrient and chemical contents. Importantly, external manure fertilizers in general contain some undesired substances, such as heavy metals or antibiotics, that are detrimental to agroecosystems. Thus, it is encouraged to apply organic manure from local agricultural ecosystems, which is assumed to be a sustainable strategy to maintain agricultural production. However, how local manure fertilizer or the combination of chemical and local manure fertilizer affects the soil nitrifying community is still poorly understood. The soil layer is another factor that shapes the nitrifier community due to the differences in nutrient and oxygen availability (Kaveney et al., 2020). The niches of canonical nitrifiers and the newly discovered comammox *Nitrospira* under different fertilization regimes and soil layers have not been extensively explored. The relationship between nitrifying microorganism abundances, community compositions and environmental factors remains to be determined.

The objectives of our study were to assess the impacts of a long-term field trial of different fertilization regimes (including chemical fertilizer, recycled manure fertilizer and chemical fertilizer combined with recycled manure fertilizer) and soil layers on the abundance, community composition and diversity of canonical nitrifiers and comammox *Nitrospira* in an Alfisol. We hypothesized that: (1) comammox *Nitrospira* are responsive to the fertilization regime and play active roles in fertilized soils due to their extensive metabolic versatility; (2) soil pH, NH_4_^+^-N and soil organic matter (SOM) contents are the most influential factors governing the niche specialization of nitrifying guilds and their relative importance to nitrification in soils; and (3) comammox *Nitrospira* clade A and clade B dominate niches in the 0–20 cm and 20–40 cm fertilized soils, respectively, due to their different ammonia affinities and different nitrogen uptake systems. The results of this study will improve our understanding of the significance of canonical nitrifiers and comammox *Nitrospira* in agricultural ecosystems and provide a robust basis for regulation strategies to improve fertilizer use efficiency in agricultural production.

## 2. Materials and methods

### 2.1. Site description and experimental design

The experimental site is located at Shenyang experimental station, Chinese Academy of Sciences, Liaoning Province, China (42°32′N, 123°23′E). The site has a temperate semihumid continental climate. The average annual temperature is 7.5°C and the mean precipitation is approximately 680 mm (Ma et al., 2020). The soil in this area is classified as an Alfisol, which is a representative soil type on the slope and piedmont plain of the Liaodong Peninsula. The formation of Alfisols is related to the soil alluvial process. Carbonates and soluble salts are leached from soil, so the leaching effect of the soil is obvious and there are many obvious rust spots in the soil profile. The soil-forming parent material is dominated by moderately acidic bedrock weathering and other non-calcareous sediments, resulting in slightly acidic to neutral soil. Due to the effects of the soil-forming process and soil parent material, the organic matter and nutrient contents in the soil are relatively high. The initial soil pH and the organic carbon, total N, P, and potassium (K) contents were 6.15, 20.9 g·kg^−1^, 1.13 g·kg^−1^, 0.44 g·kg^−1^, and 16.4 g·kg^−1^, respectively (Ning et al., 2020).

This long-term fertilization experiment consisting of 12 treatments (with three replicated plots) was established in 1990 with completely randomized block design. The area of each field plot was 162 m^2^ (9 × 18 m). Five treatments were chosen in our study, i.e., no fertilizer (CK), chemical N fertilizer (N), chemical N, P, and K fertilizers (NPK), recycled organic manure (M) and a combined application of chemical N, P, K and recycled manure (MNPK). Maize monoculture has been implemented in each treatment with three replicates since 2012. The input rates of the N, P and K fertilizers for maize were 150 kg·N·ha^−1^, 25 kg·P·ha^−1^, and 60 kg·K·ha^−1^ when respective urea, triple superphosphate and potassium chloride fertilizers were applied. One distinctive trait of this long-term experiment was that the treatments contained recycled organic manure. The recycled organic manure used in the M and MNPK treatments was from the internal nutrient recycling process under the corresponding treatments. Detailed information about the preparation of the recycled manure has been presented in previous studies (Ma et al., 2020; Ning et al., 2020). Because the application amounts of recycled manure were determined by the crop production of the previous year under the corresponding treatment, the application amounts of the recycled manure changed among different years and treatments. The input rates for each treatment in 2018 are listed in Table 1. Detailed information about the historical cultivation system, nutrient internal recycling model and fertilizer application rates is provided in the Supplementary material.

**Table 1:** Experimental design and nutrient inputs of selected treatments in 2018.

Fertilizer regimes	N (kg·ha^−1^·year^−1^)	P (kg·ha^−1^·year^−1^)	K (kg·ha^−1^·year^−1^)
CK	0	0	0
N	150	0	0
NPK	150	25	60
M^a^	(40.1)	(7.24)	(14.8)
MNPK^b^	199.9 (49.9)	41.4 (16.4)	93.0 (33.0)

a The amounts of N, P, and K in M treatment are calculated by their contents in the recycled organic manure, which are listed in the parenthesis.

b The amounts of N, P, and K in MNPK treatment are calculated by the sum of their contents in the recycled organic manure and inorganic chemical fertilizers, which are listed outside the parenthesis. The amounts of N, P and K in the parenthesis are the parts from the recycled manure.

### 2.2. Soil sampling and analysis of soil properties

Soil samples were collected on October 19, 2018 (maize was seeded on April 1st and harvested on October 18) using stainless steel samplers. It each subplot, two soil depths were sampled corresponding to the topsoil (T, 0–20 cm) and subsoil (S, 20–40 cm). We collected 30 soil samples in total and transferred them to the laboratory on ice within 2 days. One portion of the soils was sieved using 2-mm mesh and was stored at 4°C before physicochemical property analysis and another portion was stored at-80°C before DNA extraction and subsequent high-throughput sequencing analyses.

Soil pH was determined using a pH meter (1:2.5 soil/deionized water; Mettler-Toledo FiveEasy Plus, United States). The SOM content was determined using the K_2_CrO_7_ oxidation–reduction spectrophotometric method (Bao, 2000). The soil water content (SWC) was determined based on the weight loss when 2 g of fresh soil was dried to a constant weight at 105°C. Soil total P (TP) was digested with H_2_SO_4_ and HClO_4_ solution using 0.5 g of soil, and soil available P was extracted with 100 ml of 0.5 M NaHCO_3_ at a 1:20 ratio of soil to solution. A flame atomic absorption spectrophotometer (PerkinElmer 900 T-PinAAcle, MA, United States) was used to analyze the soil available K extracted with ammonium acetate. The available N in soils was determined using the Kjeldahl digestion method. The soil inorganic NO_3_^−^-N and NH_4_^+^-N contents were extracted with 2 M KCl solution and measured with a continuous flow analyzer (Futura, Alliance Instruments, Pairs, France; Bao, 2000). Soil microbial biomass C and N (SMBC and SMBN) were measured using the chloroform fumigation extraction method with minor modifications as described by Wu et al. (1990). After the soil was grinded and sieved through 100-mesh sieves, the soil total carbon (TC) and total N (TN) contents were measured by dry combustion using a Vario Max Elemental CNS analyzer (Elementar Analysesysteme GmbH, Germany).

### 2.3. Soil ammonia and nitrite oxidation potentials

Soil potential ammonia oxidation (PAO) was assessed using a modification of the method presented by Kurola et al. (2005). Five grams of equivalent dry mass of fresh soil was incubated with 20 ml of 1 mM (NH_4_)_2_SO_4_ and 10 mg·L^−1^ potassium chlorate solution (to inhibit nitrite oxidation) dissolved in phosphate buffer solution (0.2 g·L^−1^ NaH_2_PO_4_, 0.2 g·L^−1^Na_2_HPO_4_, 8.0 g·L^−1^ NaCl, 0.2 g·L^−1^ KCl, pH 7.4) for 24 h on a rotating shaker at 170 rpm and 25°C. Then, the suspension was extracted with 2 M KCl and centrifuged at 5,000 rpm for 4 min. The NO_2_^−^-N content in the filtered solution was measured with a continuous flow analyzer (Futura, Alliance Instruments, Pairs, France).

The soil potential nitrite oxidation (PNO) was measured using the method modified by Attard et al. (2010). In brief, 5.0 g of equivalent dry mass of fresh soil was agitated in 30 ml of 1.67 mg·L^−1^ NaNO_2_ solution (each gram of dry soil contained 10 μg of NO_2_^−^-N) for 15 h on a rotating shaker at 170 rpm and 25°C. The NO_2_^−^-N in the suspension was filtered and measured, and the linear decrease rates were considered PNO.

### 2.4. Soil DNA extraction and quantitative PCR assay

Soil DNA was extracted from approximately 0.5 g of fresh soil using the FastDNA Spin Kit for soil (MP Biomedicals, CA, United States) following the manufacturer’s recommendations. The DNA quality was detected by 1% (*w*/*v*) agarose gel electrophoresis and the DNA concentrations were evaluated by a NanoDrop™ One UV–Vis spectrophotometer (Nanodrop Technologies, Wilmington, United States).

The primer sets Arch-amoAF/R (Francis et al., 2005), amoA-1F/2R (Rotthauwe et al., 1997), comaA-244f/659r (a-f), and comaB-244f/659f (a-f; Pjevac et al., 2017) were used to quantify the *amoA* gene abundances of AOA, AOB, comammox *Nitrospira* clade A and comammox *Nitrospira* clade B, respectively. The *nxrA* gene of *Nitrobacter-*like NOB and the *nxrB* gene of *Nitrospira-*like NOB were amplified using the primers F1norA/R2norA (Poly et al., 2008) and nxrB169F/638R (Pester et al., 2014), respectively. Quantitative PCRs (qPCRs) were conducted using a Bio-Rad CFX384 optical real-time PCR system (Bio-Rad Laboratories Inc., Hercules, CA, United States). The 20 μL PCR mixture contained 10 μL of 2 × SYBR Premix Ex Taq™ (TaKaRa Biotechnology Co., Dalian, China), 0.8 μL of each primer (10 μM), 2 μl of DNA template (diluted 10-fold) or 2 μL of standard plasmid and 6.4 μL of dd H_2_O. Standard curves were constructed using serially diluted plasmids containing the target genes at the final concentrations of 10^8^ to 10^1^ gene copies·μL^−1^. Negative controls containing water as the template were simultaneously analyzed to eliminate any contamination. Primer set sequences and PCR thermal protocols are listed in Supplementary Table S1. All assays were performed in triplicate and the correlation coefficient (*R^2^*) values above 0.99 were accepted. To evaluate the amplification specificity of qPCR, we conducted melting curve analysis at the end of each run. The efficiencies of qPCR were 94, 101, 96, 102, 95, and 92% for AOB, AOA, comammox *Nitrospira* clade A, comammox *Nitrospira* clade B, *Nitrospira-*like NOB and *Nitrobacter-*like NOB, respectively.

### 2.5. Comammox *Nitrospira amoA* gene amplicon sequencing and phylogenetic analysis

For the high-throughput sequencing analysis, the comammox *Nitrospira amoA* gene was first amplified using the primer set ComaA189Y/ComaC576R (Xia et al., 2018). Then the PCR products were used as templates and amplified using the barcoded primer set ComaA209F/ComaC576R (Xia et al., 2018). The amplification protocols of the two rounds of nested-PCR are listed in Supplementary Table S1. After purification (QIAEX Gel Extraction Kit, Qiagen, Germany), the DNA concentrations were quantified using a Quant-iT™ PicoGreen™ dsDNA Assay Kit (Thermo Fisher Scientific, MA, United States). The purified PCR amplicons were incorporated together at equimolar concentrations and sequenced on an Illumina MiSeq platform at Majorbio (Majorbio Biopharm Technology Co., Ltd., Shanghai, China). Raw sequences were processed using Quantitative Insights in Microbial Ecology (QIIME; Caporaso et al., 2010) and Mothur (Schloss et al., 2009). Barcodes were used to split raw reads into different samples. High-quality sequences (quality score higher than 25) were retained. The obtained quality-screened sequences were divided into operational taxonomic units (OTUs) based on 97% sequence similarity for the comammox *Nitrospira amoA* gene using USEARCH (version 1.8.0). One representative sequence was chosen from each OTU for subsequent analysis. The 50 most abundant representative sequences from the remaining high-quality sequences and closely related sequences selected from the NCBI BALST database were aligned with the Clustal X program. Phylogenetic trees were constructed with neighbor-joining (NJ) method using MEGA 7.0 (Tamura et al., 2007), and bootstrap analysis was performed using 1,000 replicates. The acquired comammox *Nitrospira* sequences were deposited in GenBank with accession numbers SRR13319769 to SRR13319798.

### 2.6. Statistical analysis

The mean values and standard errors for each parameter were calculated from triplicate samples. One-way analysis of variance (ANOVA) was performed with SPSS Statistics 20.0 software (IBM, United States) based on Duncan’s test and *p* < 0.05 was considered statistically significant. Spearman’s correlation analyses were conducted to investigate the relationships among the soil physiochemical properties, potential nitrifier activities, and nitrifier functional gene abundances. Sobs, Shannon and phylogenetic diversity (PD) indices were calculated in R 3.6.0 using the ‘vegan’ package. Canonical correspondence analysis (CCA) was conducted to investigate the influence of the soil physiochemical properties on nitrifier functional gene abundances based on qPCR results and the comammox *Nitrospira* community based on high-throughput sequencing data. Permutational multivariate analysis of variance (PERMANOVA) was performed to explore the significance of the effects of the fertilization regime and soil layer on the nitrifying microbial community composition. The proportions of variations in the comammox *Nitrospira* community explained by soil properties were calculated by the “permu.hp” command in the “rdacca.hp” package (Lai et al., 2022). The Mantel test was used to identify potential environmental factors contributing to comammox *Nitrospira* community dissimilarity. Aggregated boosted tree (ABT) analysis was conducted to explore the relative contribution of soil properties to nitrifier gene abundances using the package “gbmplus” in R (Death, 2007). Structural equation modeling (SEM) was conducted with AMOS 21.0 (Amos, Development Corporation, Meadville, PA, United States) to test the hypothetical causal relationships among the fertilization regime, soil properties, soil microbial biomass (SMBC or SMBN), abundances of nitrifiers and soil nitrification potential (potential ammonia oxidation or potential nitrite oxidization). In the SEM analyses, modeling including all soil variables was conducted first to confirm which factors were significantly related to nitrifier abundances and nitrification potential (PAO or PNO) with the maximum likelihood estimation method. The adequacy of the SEM was determined by the chi-square/degrees of freedom values (CHI/DF), adjusted goodness of fit index (AGFI), root mean square error of approximation (RMSEA) index and Akaike information criteria (AIC). Favorable model fits adopted were suggested by Duan et al. (2018). Finally, we removed some non-significant variables and chosen the soil organic matter, NH_4_^+^-N, NO_3_^−^-N, total N, and available N contents as soil properties for the best model fit.

## 3. Results

### 3.1. Soil physicochemical properties

Most of the measured soil physicochemical properties were significantly affected by the fertilization regime and soil layer (*p* < 0.05; Table 2). Significant decreases in soil pH occurred in the N-treated soils compared with the CK soils, while the application of recycled manure alone had no obvious effect on the soil pH value. Among the 5 fertilization treatments, the contents of SOM, total N (TN), total P (TP), total K (TK), available N, available P, and available K reached their maximum values under the MNPK treatment, which received the highest rates of organic and chemical fertilizers. The addition of recycled manure alone also increased the SOM, soil TN, TK, TP, and available P contents to a certain degree compared with those of the CK and N treatments. Similarly, the concentrations of NO_3_^−^-N and NH_4_^+^-N were significantly increased in all fertilized treatments in comparison with those of the CK. The highest concentrations of NO_3_^−^-N and NH_4_^+^-N were found in the topsoil under the MNPK and N treatments, respectively.

**Table 2:** Basic characteristics of soil properties under different fertilization regimes and soil layers.

Soil layer (cm)	Treatment	SWC (%)**	pH**	SOM (g·kg^−1^)**	TN (g·kg^−1^)**	TP (g·kg^−1^)**	TK (g·kg^−1^)	AN (mg·kg^−1^) **	AP (mg·kg^−1^)**	AK (mg·kg^−1^)*	NH_4_^+^-N (mg·kg^−1^)**	NO_3_^−^-N (mg·kg^−1^)**	SMBC (mg·kg^−1^)**	SMBN (mg·kg^−1^)**	C/N
0–20	CK	17.55 (0.96)a	6.92 (0.05)a	16.32 (0.89)cd	0.87 (0.01)c	0.37 (0.02)d	20.10 (0.20)c	96.33 (2.52)b	3.33 (0.51)c	72.33 (3.51)bc	9.76 (0.03)d	6.07 (0.02)c	152.43 (3.32)b	21.28 (1.25)b	10.84 (0.56)a
	N	16.84 (4.02)a	5.16 (0.21)d	15.63 (0.60)d	0.90 (0.02)c	0.37 (0.00)d	20.67 (0.32)b	105.00 (2.65)b	2.13 (0.68)c	60.00 (5.29)c	13.56 (0.35)a	9.50 (0.10)b	82.48 (6.93)c	12.70 (1.73)b	10.05 (0.19)a
	NPK	17.45 (1.72)a	6.24 (0.05)b	17.03 (0.29)c	0.95 (0.02)bc	0.50 (0.01)b	20.83 (0.15)ab	103.33 (2.52)b	19.37 (2.73)b	83.33 (4.04)b	10.72 (0.57)c	6.16 (0.13)c	114.64 (18.69)bc	18.34 (4.56)b	10.36 (0.21)a
	M	18.24 (0.96)a	6.90 (0.08)a	19.24 (0.23)b	1.01 (0.05)b	0.41 (0.01)c	21.17 (0.15)a	98.33 (6.03)b	4.07 (0.23)c	75.67 (1.53)b	7.99 (0.33)e	6.12 (0.03)c	216.33 (51.44)a	30.02 (8.74)a	11.05 (0.74)a
	MNPK	17.58 (0.96)a	5.87 (0.19)c	20.40 (0.29)a	1.12 (0.0.9)a	0.60 (0.05)a	20.57 (0.31)b	123.00 (16.37)a	37.67 (1.15)a	103.00 (14.18)a	11.74 (1.00)b	21.82 (0.05)a	122.40 (11.71)bc	15.68 (2.73)b	10.60 (0.77)a
20–40	CK	21.25 (1.26)a	7.02 (0.14)a	10.98 (0.16)b	0.53 (0.03)c	0.36 (0.02)bc	21.30 (0.26)a	72.33 (6.66)a	2.63 (0.60)c	67.67 (7.57)a	5.94 (0.38)c	1.40 (0.09)d	47.08 (4.97)a	6.31 (0.50)a	12.05 (0.92)a
	N	20.78 (1.96)a	6.50 (0.21)b	10.71 (0.08)b	0.67 (0.01)a	0.34 (0.01)c	20.60 (0.20)b	69.33 (3.21)a	1.57 (0.90)c	66.67 (6.03)a	7.17 (0.06)a	4.47 (0.33)a	48.39 (9.25)a	4.01 (1.09)a	9.25 (0.21)c
	NPK	21.13 (1.56)a	6.89 (0.12)a	11.14 (0.32)b	0.67 (0.03)a	0.38 (0.02)ab	20.23 (0.32)b	72.67 (6.81)a	4.87 (1.00)b	72.67 (4.16)a	6.55 (0.15)b	2.38 (0.09)c	36.40 (8.52)a	4.31 (3.56)a	9.62 (0.55) bc
	M	20.92 (0.32)a	7.01 (0.06)a	10.95 (0.40)b	0.61 (0.02)b	0.36 (0.01)bc	20.27 (0.47)b	67.00 (4.58)a	3.13 (0.71)bc	64.67 (6.03)a	5.66 (0.09)c	1.60 (0.29)d	55.73 (9.40)a	7.38 (2.91)a	10.45 (0.58)b
	MNPK	20.93 (1.45)a	6.85 (0.13)a	12.27 (0.72)a	0.67 (0.05)a	0.40 (0.01)a	20.27 (0.06)b	72.67 (5.77)a	8.70 (1.54)a	74.00 (2.65)a	7.39 (0.31)a	3.80 (0.02)b	49.94 (19.45)a	4.87 (1.63)a	10.62 (0.63)b

The values in the parentheses present standard deviations (*n* = 3), and the different letters indicate significant differences among different treatments at *P* < 0.05 level by one-way (ANOVA). Significant difference of soil physicochemical properties between topsoil and subsoil were marked with one asterisk (*) suggesting *p* < 0.05 and two asterisks (**) suggesting *P* < 0.01 based on the Paired-sample *t*-test. SWC, soil water content; SOM, soil organic matter; TN, total N; TP, total P; TK, total K; AN, available N; AP, available P; AK, available K; NH_4_^+^-N, ammonium N; NO_3_^−^-N, nitrate N; SMBC, soil microbial biomass C; SMBN, soil microbial biomass N; C/N, the ratio of total C to total N.

Soil microbial biomass C showed a marked decrease after the application of chemical N, and the lowest content of SMBC in the topsoil was found under the N treatment, with a value of 82.48 mg·kg^−1^. Recycled manure application markedly increased the SMBC content, and the highest content was found under the M treatment. Although the nutrient inputs of the MNPK treatment were the highest, the SMBC under the MNPK treatment was less than that under the M treatment, which showed the negative effects of chemical N fertilizer on the SMBC. A similar pattern was found for SMBN, and the highest amount of SMBN also appeared under the M treatment. There was no significant difference in the C/N ratio of the topsoil among the different treatments. Unsurprisingly, the SOM, TN, TP, available N, available P, available K, NH_4_^+^-N and NO_3_^−^-N contents of the subsoil were significantly lower than those of the topsoil (Table 2). Conversely, SWC and pH in the subsoil were higher than those in the topsoil.

### 3.2. Soil potential ammonia oxidation and nitrite oxidation activity

**Figure 1:**
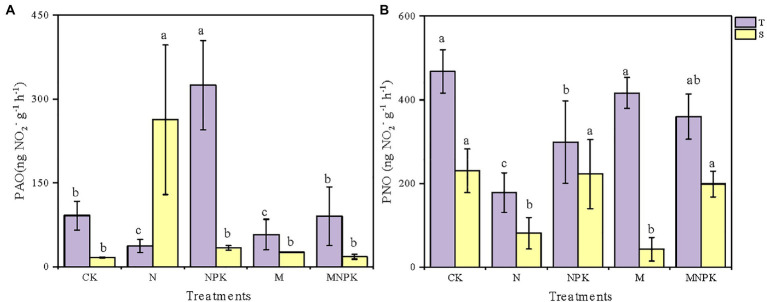
Soil potential ammonia oxidation (PAO). **(A)** Potential nitrite oxidation (PNO). **(B)** Under different fertilization regimes in the two soil layers. Values are shown as mean ± standard deviations (*n* = 3). Different letters above the bars indicate significant difference among fertilization regimes using the Duncan multiple-range test. T, topsoil 0–20 cm; S, subsoil 20–40 cm.

Both soil PAO and PNO varied significantly among soil samples from the different fertilization regimes and soil layers (Figure 1; Supplementary Table S2). Generally, due to the higher nutrient and oxygen contents in the surface soil, the soil PAO and PNO of the topsoil were higher than those of the subsoil under the same fertilization regime except for the soil PAO under the N treatment. In the topsoil, the PAO varied from as low as 16.53 (±0.71) to 324.80 (±79.90) ng·NO_2_^−^·g^−1^·h^−1^ (Figure 1A) and peaked under the NPK treatment, which was 2.6 times higher than that under the CK. The soil PAO under the CK and MNPK treatments was comparable. Unexpectedly, the lowest PAO of the topsoil and the highest PAO of the subsoil both occurred under the N treatment.

The responses of the soil PNO to the fertilizer regime were different from the changes in the soil PAO (Figure 1B). In the topsoil, the CK treatment had the highest PNO of approximately 468.13 (±51.28) ng·NO_2_^−^·g^−1^·h^−1^, while the N treatment had the lowest PNO of 178.53 (±47.27) ng·NO_2_^−^·g^−1^·h^−1^. The soil PNO under the recycled manure treatment was slightly lower than that under the CK treatment but was as much as 2.33 times higher than that under the N treatment. The measured soil PNO of the subsoil ranged from 43.07 (±28.28) to 230.47 (±52.38) ng·NO_2_^−^·g^−1^·h^−1^, which was significantly lower than that of the topsoil.

### 3.3. Abundances of canonical ammonia oxidizers, comammox *Nitrospira* and nitrite oxidizers

**Figure 2:**
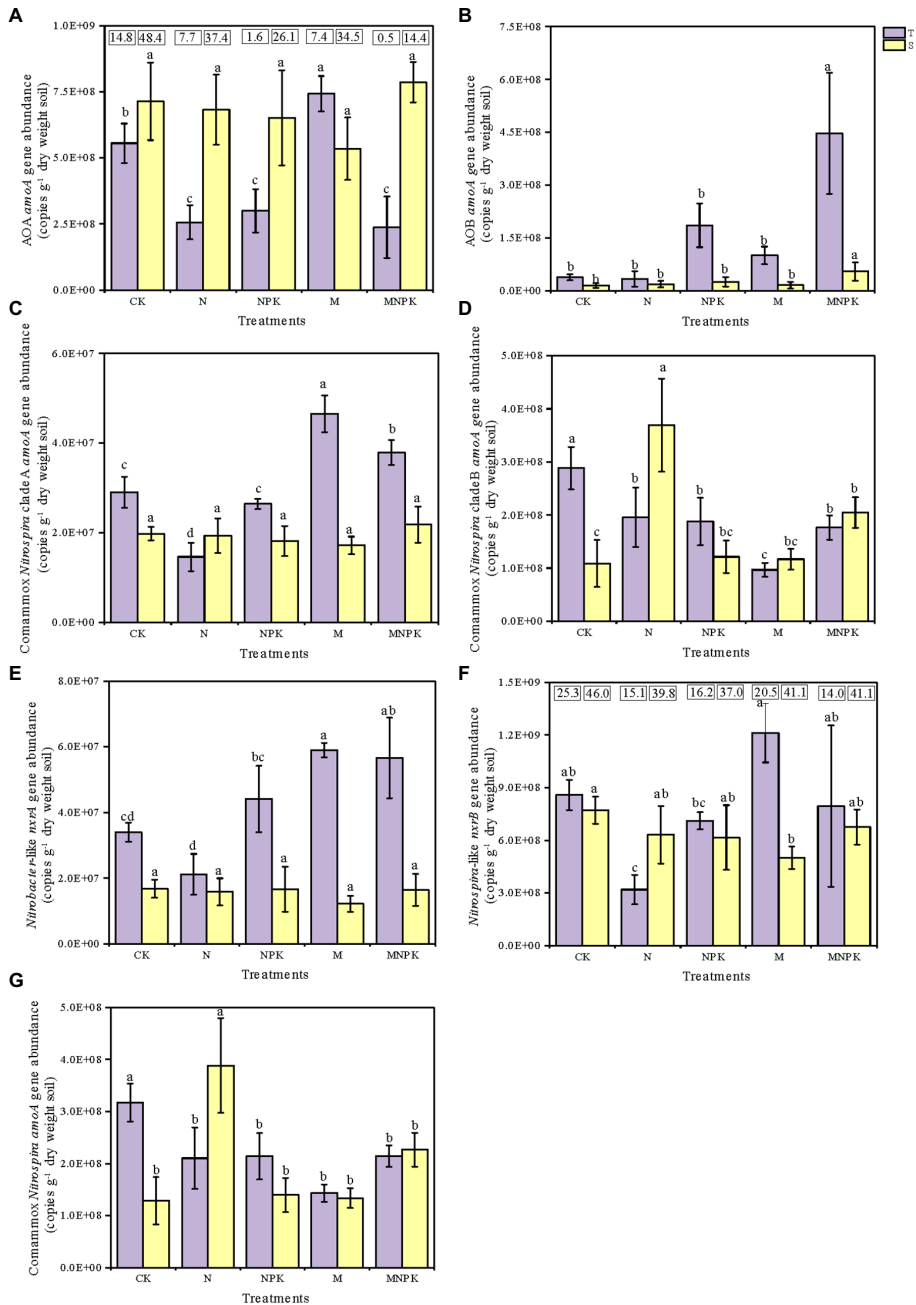
Abundances of nitrifier genes under different long-term fertilization regimes. The *amoA* gene abundances of AOA **(A)**, AOB **(B)**, comammox *Nitrospira* clade A **(C)**, comammox *Nitrospira* clade B **(D),** total comammox *Nitrospira* (the sum of clade A and clade B *amoA* gene abundances) **(G)**, the *nxrA* gene abundance of *Nitrobacter*-like NOB **(E)**, and the *nxrB* gene abundance of *Nitrospira-like* NOB **(F)** were determined by qPCR analysis. The numbers in the panel **(A,F)** express the ratio of AOA to AOB and *Nitrospira* to *Nitrobacter*. The error bars in the columns represent the standard errors of the triplicated means. The different letters above columns indicate significant differences among different treatments (one-way ANOVA, Duncan test, *P*<0.05). T and S represent topsoil and subsoil, respectively.

Quantitative PCR (qPCR) analysis was adopted to quantify the *amoA* gene abundances of AOB, AOA, and comammox *Nitrospira* clade A and clade B in response to the fertilization regime and soil layer (Figures 2A–D,G). The results showed that the long-term fertilization regime significantly changed the *amoA* gene abundances of AOA and AOB in the soil (Supplementary Table S2; Figure 2). In the topsoil, the AOA *amoA* gene abundance significantly increased under the M treatment, but significantly decreased with the application of chemical N fertilizer. In contrast, the AOB *amoA* gene abundance demonstrated an increasing tendency accompanied by increasing rates of nutrients, and the highest *amoA* gene abundance of AOB was found under the MNPK treatment, which was as much as 11.88 times greater than that under the CK. Across all of the treatments, the *amoA* gene abundances of AOA ranged from 2.37 × 10^8^ to 7.86 × 10^8^ copies g^−1^ dry soil, which were maximum 48.4 times greater than those of AOB, which ranged from 1.48 × 10^7^ to 4.47 × 10^8^ copies g^−1^ dry soil (Figures 2A,B). Moreover, the *amoA* gene abundances of AOA and AOB displayed opposite responses to different soil depths.

Similar to AOA, the comammox *Nitrospira* clade A *amoA* gene abundances were considerably increased by the application of recycled manure (Figure 2C). The highest abundance of comammox *Nitrospira* clade A (4.65 × 10^7^ copies per grams of dry soil) occurred in the topsoil under the M treatment. Significant differences were detected in the comammox *Nitrospira* clade A abundance between the two soil layers (Supplementary Table S2), and the abundances of comammox *Nitrospira* clade A decreased with increasing soil depth under the CK, NPK, M, and MNPK treatments. The abundance of comammox *Nitrospira* clade B was much higher than that of comammox *Nitrospira* clade A in all soil treatments, ranging from 9.68 × 10^7^ to 3.69 × 10^8^ copies g^−1^ dry soil (Figure 2D). Among all treatments, the highest comammox *Nitrospira* clade B abundance occurred in the subsoil under the N treatment. There was an increasing trend in the comammox *Nitrospira* clade B abundance with increasing soil depth except in the CK and NPK treatments. Additionally, the total comammox *Nitrospira* abundances (the sum of the *amoA* gene abundances of clade A and clade B) ranged from 1.29 × 10^8^ to 3.88 × 10^8^ copies g^−1^ dry soil, this range was slightly lower than that of AOA (Figure 2G).

The *Nitrobacter*-like *nxrA* gene and *Nitrospira*-like *nxrB* gene in all soil samples ranged from 1.22 × 10^7^ to 5.90 × 10^7^ and 3.20 × 10^8^ to 1.21 × 10^9^ copies g^−1^ dry soil, respectively (Figures 2E,F). Manure fertilizer markedly increased the NOB abundance in contrast with the effects of chemical N fertilizer. Significantly higher abundances of the *Nitrobacter nxrA* gene and the *Nitrospira nxrB* gene were found in the topsoil under the M treatment, 2.8 and 3.8 times higher, respectively, than those under the N treatment.

### 3.4. Soil properties influencing the distribution of nitrifier gene abundances

**Figure 3:**
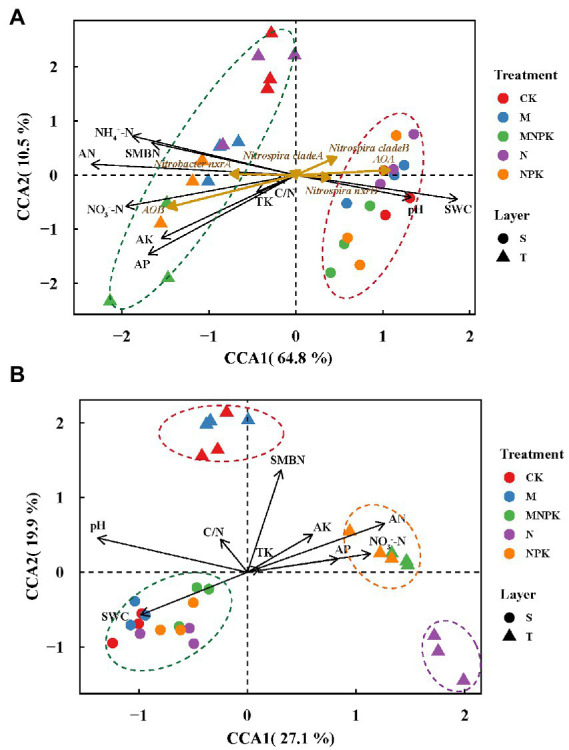
Canonical correspondence analysis (CCA) plot based on the gene abundances of all nitrifying populations **(A)** and comammox *Nitrospira* OTU compositions **(B).** Circles, subsoil samples, S; triangles, topsoil samples, T; black arrows, soil physiochemical properties; dark goldenrod arrows, nitrifiers. Red, blue, green, purple, and orange colors represent CK, M, MNPK, N and NPK treatments, respectively. Abbreviations: SWC, soil water content; TK, total K; AN, available N; AP, available P; AK, available K; NH_4_^+^-N, ammonium N; NO_3_^−^-N, nitrate N; SMBN, soil microbial biomass N; C/N, the ratio of total C to total N.

Canonical correspondence analysis (CCA) was conducted to elucidate variances in the nitrifying guild abundances among different treatments and their responses to the soil properties. The first two CCA dimensions explained 75.3% of the cumulative variations and soils were assembled by layers (Figure 3A). The results showed that AOB and *Nitrobacter*-like NOB were more abundant in the topsoil with higher soil nutrient contents and SMBN, while AOA, *Nitrospira*-like NOB and comammox *Nitrospira* clade B tended to be more abundant in the subsoil with a higher pH and soil water content.

Aggregated boosted tree analysis was conducted to elucidate the importance of soil properties in predicting the changes in nitrifier gene abundances (Supplementary Figure S1). The results suggested that different soil properties contributed differently to the variations in the nitrifier gene abundances. The two most crucial soil properties driving the AOA abundances were soil pH (24.3%) and NH_4_^+^-N (18.3%). SOM content was the most important contributor to the changes in AOB (21.7%), comammox *Nitrospira* clade A (36.2%), *Nitrobacter*-like NOB (37.9%) and *Nitrospira-like* NOB (16.5%) abundances in all samples. The contribution of the soil available P content (17.2%) to the comammox *Nitrospira* clade B abundances was predominant. Overall, among the determined soil physiochemical properties, the soil pH, NH_4_^+^-N, soil organic matter, and available P content were the principal factors that shaped the nitrifier abundances in the fertilized soils.

### 3.5. Structural equation modeling analysis

**Figure 4:**
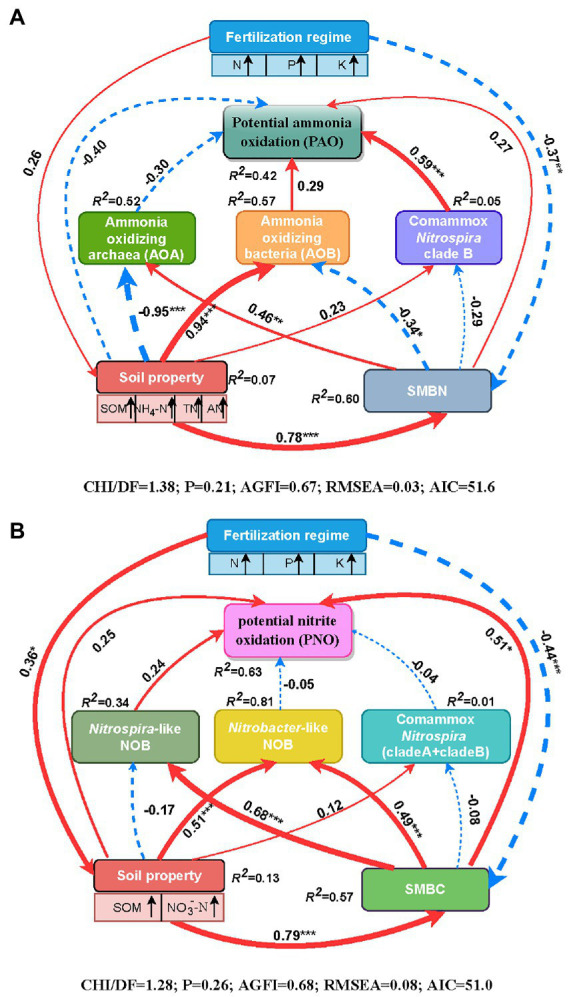
Path diagrams demonstrating the potential causal relationships among the fertilization regime, soil properties (SOM, NO_3_^−^-N, NO_4_^+^-N, TN and available N), soil microbial biomass (C and N), the abundances of nitrifiers (AOA, AOB, comammox *Nitrospira* clade A, comammox *Nitrospira* clade A, *Nitrobacter*-like NOB and *Nitrospira* like NOB) and soil PAO **(A)** or PNO **(B)**. The width of the arrows in proportional to the strength of the potential causal effects adjacent with path coefficients. The red solid and blue dashed lines indicate the positive and negative relationships between the indicators, respectively. The *R^2^* demotes the percentage of variance explained by the corresponding indicator in the structural equation modeling. Significant levels are indicated as follows: *p < 0.05, **p < 0.01, and ***p < 0.001. SOM, soil organic matter; TN, total N; AN, available N; NH_4_^+^-N, ammonium N; NO_3_^−^-N, nitrate N; SMBC, soil microbial biomass C; SMBN, soil microbial N.

Spearman’s correlation analysis demonstrated that soil PAO was markedly and positively correlated with the abundances of AOB, comammox *Nitrospira* clade B and total comammox *Nitrospira*. Additionally, there were significant and positive relationships between the abundances of AOB, comammox *Nitrospira* clade A, *Nitrobacter*-like NOB, *Nitrospira*-like NOB and soil PNO (Supplementary Table S3). Path analysis was conducted to further assess the direct or indirect relationships among the fertilization regime, soil properties, SMBC or SMBN, the abundances of active nitrifiers (AOA, AOB, comammox *Nitrospira* clade A, comammox *Nitrospira* clade B, *Nitrobacter*-like NOB, and *Nitrospira*-like NOB) and the soil nitrification potential (PAO and PNO) through structural equation pattern (Figure 4). With respect to soil PAO, the fertilization regime directly affected the soil properties (e.g., soil organic matter, NO_4_^+^-N, TN, and available N) through a positive path (λ = 0.26, *p* > 0.05) and significantly affected SMBN through a negative path (λ = −0.37, *p* < 0.01; Figure 4A). The comammox *Nitrospira* clade B abundance had a direct positive impact on the soil PAO (λ = 0.59, *p* < 0.001) and was affected by the negative effects of SMBN (λ = −0.29, *p* > 0.05), followed by the positive effects of the soil properties (λ = 0.23, *p* > 0.05). The AOB abundance also had a direct effect on soil PAO (λ = 0.29, *p* > 0.05), but the positive effect of AOB on soil PAO was less than that of comammox *Nitrospira* clade B. Moreover, soil properties (λ = −0.40, *p* > 0.05) and SMBN (λ = 0.27, *p* > 0.05) were directly related to soil PAO, although the effects were not significant. Overall, 42% of the variability in soil PAO could be explained by the parameters (*R^2^* = 0.42 in Figure 4A). In addition, the results of the standardized total effects revealed that the abundance of comammox *Nitrospira* clade B had the strongest positive effects on soil PAO (Supplementary Figure S2).

With respect to soil PNO, the fertilization regime directly affected the soil properties (e.g., soil organic matter and NO_3_^−^-N) through a positive path (λ = 0.36, *p* < 0.05) and significantly affected SMBC through a negative path (λ = −0.44, *p* < 0.001; Figure 4B). Both the soil properties (λ = 0.24, *p* > 0.05) and SMBC (λ = 0.51, *p* < 0.05) affected soil PNO directly, and they also affected soil PNO indirectly *via* the influence on the nitrifier abundance. The direct relationship between PNO and the abundances of *Nitrospira*-like NOB was positive but not significant (λ = 0.24, *p* > 0.05). Overall, the model explained 63% of the variation in soil PNO (*R^2^* = 0.63, Figure 4B). Furthermore, the standardized total effects showed that the soil properties and SMBC were important factors, as the soil properties had the strongest indirect effects on soil PNO, and SMBC had the strongest direct effect on the PNO (Supplementary Figure S2).

### 3.6. Phylogeny of comammox *Nitrospira*

A total of 2,053,861 high-quality sequences were retrieved from the 30 soil samples. According to the cutoff threshold of 97% nucleotide similarity, 246 unique OTUs were identified. A phylogenetic tree containing the 50 major OTU sequences (the proportion > 0.05) accounting for more than 98.9% of the total OTU abundances was constructed using the neighbor-joining method with the p-distance model. Consistent with previous observations, the comammox *Nitrospira amoA* gene phylogenetic tree showed that the major OTU sequences were clustered into two clades, where 18 out of the 50 sequences were affiliated with comammox *Nitrospira* clade A within clade A2. The clade A2 cluster was affiliated with the metagenome-assembled genome (MAG) species *SG-bin1* (17 OTUs) and *SG-bin2* (1 OTU). There were 21 OTUs belonging to clade B (Supplementary Figure S3), affiliated with *Nitrospira* sp. *CG24C and Nitrospira sp*. *CG24A*. Notably, some of the selected-OUT clusters (11 out of 50) were close to those of the AOB *amoA* sequences due to the nonspecific amplification of the selected comammox *Nitrospira amoA* gene primer set and partial nested PCR protocol (Supplementary Figure S3). Among the 50 most abundant OTUs, comammox *Nitrospira* clade B (42%) was the dominant cluster compared with clade A (36%; Supplementary Figure S3).

**Figure 5:**
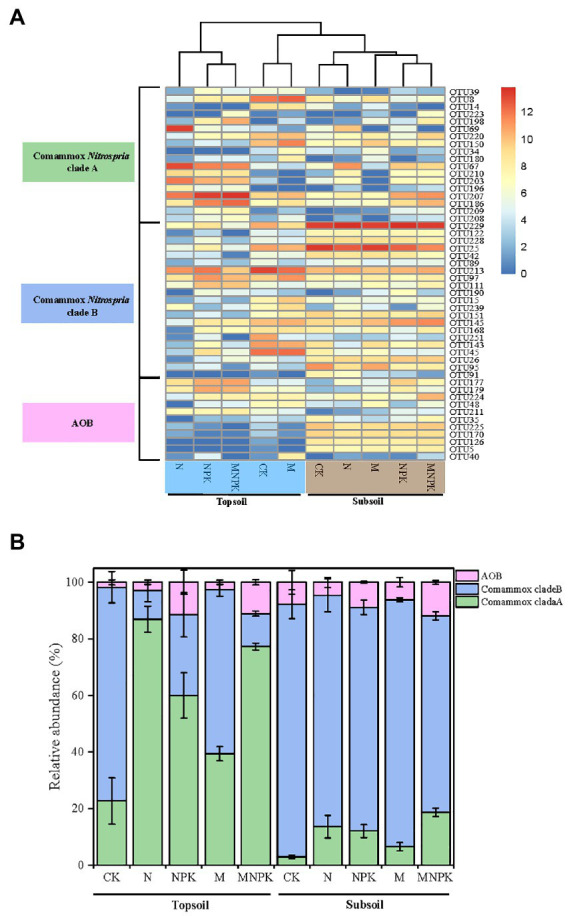
Heatmap plotting the relative abundance of the 50 major OTUs (relative abundance > 0.05 in at least one sample) **(A)** and community composition of comammox *Nitrospira* based on *amoA* gene sequences **(B)** retrieved for the 30 soil samples. Error bars are standard error of means (*n* = 3).

Cluster analysis showed that the distribution of OTUs belonging to clade A and clade B varied obviously among the different soil treatments (Figure 5A). The topsoil under the CK and M treatments clustered closely with the subsoil. Meanwhile, the relative abundances of clade A and clade B responded differently to the fertilization regime and soil layer (Figure 5B). Clade A was abundant in the topsoil and dominated under the N and MNPK treatments, making up 86.5 and 76.4%, respectively, of the selected OTUs. In contrast, although clade B seemed to be dominant in the topsoil under the CK (74.3%) and M (56.6%) treatments, the relative abundance of clade B was higher in the low nutrient-replenished subsoil (ranging from 78.7 to 88.1%), indicating niche differentiation between comammox *Nitrospira* clade A and clade B. The highest proportion was found in the subsoil under CK (88.1%), suggesting the preference of clade B in oligotrophic environments.

### 3.7. Soil properties shaping the community of comammox *Nitrospira*

Canonical correspondence analysis was performed to estimate the main soil properties affecting the community structure of comammox *Nitrospira* under different treatments, and the first two CCA dimensions explained 47.0% of the cumulative variation in the comammox *Nitrospira* community compositions (Figure 3B). Notably, pH, SMBN and available N explained 27.1, 17.7 and 14.5%, respectively, of the whole community dissimilarity (*p* < 0.01), acting as the three most critical factors. Soil samples from the subsoil were closely grouped and obviously differed those from the topsoil. PERMANOVA further demonstrated that the comammox *Nitrospira* community composition not only significantly diverged with the soil layer (*R^2^* = 0.41, *p* = 0.001), but also with the fertilization regime (*R^2^* = 0.30, *p* = 0.001). The compositions of comammox *Nitrospira* in the topsoil were divided into three groups depending on the fertilization regime. The ranking of the Bray-Curtis distances revealed that there were obvious differences in the comammox *Nitrospira* communities among the different fertilization treatments in the topsoil (Figure 3B; Supplementary Figure S4). Pearson correlation analysis revealed that the Sobs index of the comammox *Nitrospira* community (alpha diversity) showed significant positive correlations with pH (*r* = 0.44, *p* < 0.05) and the soil water content (*r* = 0.42, *p* < 0.05), but showed marked negative correlations with available N (*r* = −0.37, *p* < 0.05) and NH_4_^+^-N (*r* = −0.37, *p* < 0.01; Table 3). The Mantel test revealed that the dissimilarity in the beta diversity of comammox *Nitrospira* composition was significantly correlated with pH and the soil organic matter, TN, available N, NH_4_^+^-N and NO_3_^−^-N contents (Table 3). Considering the influence of soil properties on the abundance and composition of comammox, we confirmed that soil pH and the NH_4_^+^-N and soil organic matter contents were the most crucial factors driving the comammox *Nitrospira* community compositions.

**Table 3:** Correlations between soil properties and comammox *Nitrospira* community diversity in fertilized soils.

Factors	Comammox *Nitrospira* community				
	*α*-Diversity			*β*-Diversity	
	(Pearson correlation)			(Mantel test)	
	Sobs	Shannon	PD	Jaccard	Bray-Curtis
SWC	0.42*	−0.12	0.56**	0.40**	0.35**
pH	0.44*	0.31	0.41*	0.41**	0.54**
SOM	−0.27	0.34	−0.76**	0.38**	0.66**
TN	−0.34	0.25	−0.78**	0.34**	0.62**
TP	0.06	0.24	−0.52**	0.17	0.36**
TK	−0.14	0.07	−0.02	0.01	0.01
AN	−0.37*	0.15	−0.71**	0.34**	0.63**
AP	0.13	0.15	−0.48**	0.15	0.29**
AK	0.15	0.31	−0.39*	0.21	0.33**
NH_4_^+^-N	−0.53**	−0.10	−0.67**	0.46**	0.69**
NO_3_^−^-N	−0.12	0.02	−0.57**	0.30*	0.41**
SMBC	−0.17	0.49**	−0.55**	0.19*	0.49**
SMBN	−0.22	0.45*	−0.56**	0.20	0.43**
C/N	0.19	0.28	0.06	0.06	−0.05

Significant correlations (*p* < 0.05) are indicated in bold. *p < 0.05, **p < 0.01.

Sobs, the observed richness described the community richness; Shannon, the Shannon diversity index described the community diversity; PD, the phylogenetic diversity described the community diversity; Jaccard, transformed data by Jaccard distance; Bray-Curtis, transformed data by Bray-Curtis distance.

## 4. Discussion

With the discovery and prevailing occurrence of comammox *Nitrospira* in engineered, coastal and terrestrial environments (Li et al., 2022; Zhu et al., 2022), our previous knowledge about the niche differentiation of different nitrifiers and their relative contribution to nitrification in agricultural ecosystems need to be reconsidered. Distinct fertilization effects on canonical nitrifier abundance and community structure have been reported in many previous studies (He et al., 2007; Wang et al., 2019; Lin et al., 2020; Cai et al., 2021), and our study further confirmed this information. The abundance of AOB was enhanced by the application of chemical N fertilizer, while the changes in the abundance of AOA displayed an opposite pattern, suggesting that the addition of chemical fertilizer created an unfavorable condition for AOA (Figure 2). Compared with chemical fertilizer, recycled manure fertilizer significantly increased the abundance of AOA (Figure 2). These distinct responses of AOB and AOA to fertilization regimes can be explained by their different affinities for ammonia substrates (Prosser and Nicol, 2012; Ouyang et al., 2018). The theoretical ammonia concentration in the soil aqueous solution based on the ionization equilibrium ranged from 400 to 14,270 nM (Supplementary Table S4), which facilitated the growth of both AOA and AOB with optimal survival ammonia concentrations ranging from 0.007 to 1,220 nM and 6,730 to 323,000 nM, respectively (He et al., 2012; Hu et al., 2014). Obviously, AOB are better adapted to soils with relatively high ammonia concentrations due to their higher maximum activity and half-saturation constant value for ammonia than AOA (Hu et al., 2014; Li et al., 2022). ABT analysis demonstrated that AOB abundance was positively correlated with the soil TN and NH_4_^+^-N contents, while AOA abundance was significantly and negatively correlated with the NH_4_^+^-N content (Supplementary Figure S1). Thus, AOB were more abundant in the 0–20 cm layer of fertilized soils and peaked under the MNPK treatment receiving the highest fertilizer inputs, while AOA preferred oligotrophic environments such as unfertilized soils and subsoils (Figure 2). Notably, long-term manure recycling significantly boosted the AOA abundance. Since manure only contained a small percentage of N in urea or inorganic N form, most of the N was organically derived (Sun et al., 2021), and the negative effect of manure on the AOA abundance was slight. Some AOA species (e.g., *Nitrosophaera viennensis* and *Nitrosopumilus martimus* SCM1) are considered to be mixotrophic and possess genes encoding enzymes for assimilating organic compounds (Tourna et al., 2011). Additionally, another study supported the idea that some AOA can use certain organics to act as chemical scavengers to detoxify intracellular H_2_O_2_ rather than assimilate organics into their membrane lipids (Kim et al., 2016). Therefore, the AOA abundance was significantly stimulated by recycled manure application.

Similarly, *Nitrobacter*-like and *Nitrospira*-like NOB have different lifestyle strategies. *Nitrobacter*-like NOB are known as *r*-strategists, preferring higher N availability and oxygen concentrations (Nowka et al., 2014), whereas *Nitrospira*-like NOB are *K*-strategists, dominating in N-limited environments with higher N substrate affinity (Schramm et al., 1999). Our results supported the idea that the niche differentiation of these two genera of NOB showed that *Nitrobacter*-like NOB were more abundant in the topsoil with high nutrient contents and were more associated with AOB (Figure 3A). The application of recycled manure provided a more stable nutrient supply and increased the soil organic carbon availability and microbial biomass (such as SMBC and SMBN), thereby providing rich and balanced nutrients for both *Nitrobacter*-like and *Nitrospira*-like NOB to grow. In fact, NOB should not be considered obligate chemolithoautotrophs, and some NOB species have been proven to be capable of assimilating simple organic compounds, such as glycerol, formate and pyruvate (Daims et al., 2001; Spieck et al., 2006). Consequently, soil samples under the M treatment significantly increased the *Nitrobacter nxrA* and *Nitrospira nxrB* abundances (Figure 2).

It is well-known that comammox *Nitrospira* are responsive to nitrogen addition in soils because nitrogen as the essential substrate may shape the ecological niche differentiation of comammox *Nitrospira* in agricultural soils (Shi et al., 2018; He et al., 2021). However, knowledge of the effect of different fertilization regimes on comammox *Nitrospira* abundance and community composition is still limited. Our study showed that the abundance of comammox *Nitrospira* (the sum of clade A and clade B) was slightly higher than that of canonical AOB (except in the topsoil under MNPK) and slightly lower than that of AOA (Figure 2), suggesting that comammox *Nitrospira* might be competitively superior in fertilized Alfisols. This means that comammox *Nitrospira* not only have an oligotrophic lifestyle but can also adapt to various environments with abundant substrates (Li et al., 2020; Lu et al., 2020; He et al., 2021), which is consistent with the idea that comammox in terrestrial ecosystems might have evolved ecological features that are more diverse than those in aquatic ecosystems (Hu and He, 2017; Hu et al., 2021).

Both comammox *Nitrospira* clade A and clade B were observed in the studied soils. Although comammox *Nitrospira* clade A was proposed to be split into clades A1, A2 and A3 (Xia et al., 2018; Li et al., 2020), only comammox *Nitrospira* clade A2 was found in the studied soils (Supplementary Figure S3). Unlike the oligotrophic lifestyle of clade A1 (Xia et al., 2018; Sun et al., 2020), clade A2 was dominant in N-rich tidal flat sediment or agricultural soils (Xia et al., 2018; Liu et al., 2020; Xu et al., 2020). Specifically, a recent study demonstrated that there were different ecological preferences in individual subclades (clade A2.1 and clade A2.2) of clade A2 in Ultisols following long-term fertilization (Lin et al., 2022). These findings provide insights that comammox *Nitrospira* clade A in agricultural soil is not strictly oligotrophic and has adapted to thrive in a wide range of ecological niches. Additionally, comammox *Nitrospira* clade B, which is oligotrophic, was also found in these fertilized soils. Comammox *Nitrospira* clade A and clade B abundances responded differently to chemical and/or manure fertilizer addition. Compared with CK and NPK, the application of NPK plus recycled manure (MNPK) or recycled manure alone (M) significantly increased the abundance of comammox *Nitrospira* clade A. This is consistent with a recent study that provided evidence that comammox *Nitrospira* clade A might be relatively copiotrophic and thus flourished in NPK plus pig manure treatment with a higher nutrient content (Lin et al., 2022). Generally, comammox *Nitrospira* clade B is considered oligotrophic and sensitive to the soil NH_4_^+^-N content, so clade B did not grow autotrophically when ammonium was added to the soil (Daims et al., 2015; Shi et al., 2018; Wang et al., 2019). Therefore, the application of fertilizer, regardless of chemical fertilizer or recycled manure, decreased the abundance of comammox *Nitrospira* clade B in all topsoil samples (Figure 2). As shown in Figure 3B, the different long-term fertilization regime also led to marked differences in the community composition of comammox *Nitrospira* especially in the topsoil. The community structure of comammox under the CK and M treatment was similar and obviously different from that in the treatments containing chemical N, indicating that N forms and contents induced by the fertilization regime were key factors driving the comammox *Nitrospira* community (Sun et al., 2021; He et al., 2022).

In addition, different types of comammox *Nitrospira* had unique response patterns to soil layer. The relative abundance of comammox *Nitrospira* clade A was predominant in the topsoil especially under the chemical fertilizer treatments. In contrast, the relative abundance of clade B appeared to be dominant and increased in the subsoil samples with lower ammonium content (Figures 2, 5). This finding consistently supported the idea that there was niche separation between comammox *Nitrospira* clade A and clade B owing to their physiological differentiation and genome characteristics. Comammox *Nitrospira* clade A possesses Rh-type ammonium transporters with a higher uptake capacity and lower affinity for ammonia (homologous Rh-type ammonium transporters discovered in most β-AOB, K_m*(NH3)*_ ≈ 0.14–0.19 mM) than Amt-type ammonium transporters (Dytczak et al., 2008; Daims et al., 2015; Palomo et al., 2018). Comammox *Nitrospira* clade B encodes Amt-type ammonium transporters (present in canonical AOA, K_m*(NH3)*_ ≈ 0.13 μM) with a higher affinity for ammonia (Martens-Habbena et al., 2009; Kits et al., 2017; Koch et al., 2019). In summary, this evidence shows that comammox *Nitrospira* clade A (mainly clade A2) prefers soil that is relatively rich in ammonium and nutrients, while comammox *Nitrospira* clade B prevalently occurs in nutrient-limited soils. Different clades of comammox *Nitrospira* occupied distinct ecological niches and adaptively responded to long-term fertilization in agricultural soils, which supports our hypothesis.

Comammox *Nitrospira* can perform both ammonia-oxidation and nitrite-oxidation in the nitrification process, suggesting that they may compete with canonical nitrifiers for the transformation of nitrogen in agroecosystems. Some studies have shown that comammox *Nitrospira* demonstrated lower activity and less contribution to ammonia oxidation than canonical ammonia oxidizers (Shi et al., 2018; Wang et al., 2019, 2020). Comammox *Nitrospira* clade B was assumed to contribute more to nitrification in soil without NH_4_^+^ addition (Wang et al., 2019). However, as revealed by Spearman’s correlation and structural equation modeling analysis, our study revealed that the contributions of comammox *Nitrospira* clade B and AOB to the soil PAO were positive and significant, and canonical NOB (mainly *Nitrospira*-like NOB) were the dominant contributors to the soil PNO (Figure 4; Supplementary Table S3). These results confirmed that AOB and comammox *Nitrospira* might play crucial roles in the nitrification of agricultural soils, as previously reported (Hu et al., 2021). The reason for the important contribution of comammox *Nitrospira* clade B to soil PAO could be as follows: First, the physiological adaptation of comammox *Nitrospira* clade B might be more diverse than we previously thought; some clade B specimens found in agricultural soil might adapt well to relatively high ammonia availability in soil (He et al., 2022; Lin et al., 2022), so the high abundance of clade B in the studied soils indicated its importance in soil nitrification. Second, Liu et al. (2020) demonstrated that comammox *Nitrospira* clade B made greater contributions to the nitrifier abundance on the Qinghai-Tibetan Plateau with a higher pH and lower temperature (9.6 ± 0.4°C). The average annual temperature of experimental station was 7.5°C, which might be a favorable temperature for clade B to grow but could generate selective pressure for the other ammonia oxidizers. Third, although canonical ammonia oxidizers and comammox *Nitrospira* had the same ammonia oxidation function, they might cooperatively coexist rather than be competitively exclusive due to their niche separation (Wang et al., 2019). It is probable that AOB played an important role in the top layer of soil amended with chemical N fertilizer, and comammox *Nitrospira* clade B preferred to utilize ammonium from mineralized organic N in the subsoil. These two nitrifiers might work cooperatively and occupy different niches to perform ammonia oxidation in long-term chemically and/or manure-fertilized soils. These results support our hypothesis that comammox *Nitrospira* play an important role in soil ammonia oxidation even with high nutrient inputs. Additionally, it should be noted that the substrate addition method was adopted to measure PAO and PNO in soil, which might not accurately reflect the nitrifier *in situ* rates in soils but only their potential ability (Wang et al., 2019). The active nitrifying guilds responsible for PAO and PNO might be entirely distinct with those under *in situ* soil conditions. It is thus important to make presumptions for correlating the manipulated PAO and PNO activities with *in situ* soil microbial diversity and ability by Spearman correlation analysis alone. Moreover, techniques simultaneously combining with selective inhibitors with DNA/RNA-stable isotope probing should be adopted to determine the actual activity and contribution of comammox *Nitrospira* in agricultural soils.

In our study, the measured soil physicochemical properties were significantly affected by the fertilization regime (*p* < 0.05; Table 2). It is reported that the application of organic manure can effectively preserve soil carbon and nitrogen pools and buffer soil acidification (Xie et al., 2021), while repeated application of chemical N fertilizer can significantly decrease the soil pH (Shi et al., 2018). Notably, compared with CK, the decrease of soil pH in the topsoil under chemical NPK treatment was less than that under chemical N treatment. One possible reason for this phenomenon was that the chemical P fertilizer used in NPK treatment was calcium triple superphosphate containing a large amount of calcium cations, which improved the soil ion exchange capacity and decreased the soil pH reduction caused by chemical N fertilizer. Another reason was that the crop yield, root biomass and root exudates under NPK treatment were much higher than those under N treatment (Ning et al., 2020), which promoted the amount of organic matter returned through root stubble under NPK treatment. The higher organic matter content in soil under NPK treatment (Table 2) was conducive to improving soil buffering capacity and alleviating soil acidification.

Several studies have pointed out that environmental discrepancies and biological reciprocal actions are the key factors controlling the variations in the microbial community composition in diverse ecosystems (Bahram et al., 2018; Vijayan et al., 2020). As revealed by CCA and ABT analysis (Figure 3; Supplementary Figure S1), soil pH, NH_4_^+^-N and SOM contents were crucial factors shaping nitrifying microbial abundances and comammox *Nitrospira* communities in soils subjected to long-term fertilization. The vital roles of soil pH and ammonium in controlling the biogeographic distribution, community composition and niche separation of canonical ammonia oxidizers have been thoroughly discussed in many previous studies (He et al., 2012; Prosser and Nicol, 2012; Hu et al., 2013). Zhu et al. (2022) also found that pH was the most important factor affecting comammox *Nitrospira* abundance on a global scale. Meanwhile, ammonium concentration was identified as the dominant predictor of the variation in comammox *Nitrospira* abundance and community composition, although the effects of ammonium were inconsistent (Wang et al., 2019; He et al., 2021; Zhu et al., 2022). The reason for the different sensitivities of AOB, AOA and comammox *Nitrospira* to soil pH could be attributed to their different affinities for NH_3_ (Hu and He, 2017; Kits et al., 2017). It is well accepted that the ammonia oxidizers prefer to metabolize NH_3_ substrates rather than NH_4_^+^ (Hu and He, 2017; Xu et al., 2020). Variations in soil pH under long-term fertilization governed the nitrifier abundances because of the pH-dependent chemical equilibrium between NH_3_ and NH_4_^+^-N. The results showed that the abundance of comammox *Nitrospira* was significantly and negatively correlated with soil pH (Supplementary Figure S1), which is consistent with many studies suggesting that comammox *Nitrospira* exhibits a higher resistance to slightly acidic soils (Shi et al., 2018; Xu et al., 2020; Hu et al., 2021). The recycled manure treatment was the highlight of this long-term field trial, as all of the organic fertilizer applied in this experiment was derived from the ecosystem and then returned to the system itself as a nutrient supply. This procedure was obviously different from employing commercial organic fertilizer. First, the organic fertilizer and nutrients were all from the system itself and depended on the biological mass product. Second, the amount of organic fertilizer varied from year to year. Ultimately, although the input rates of N, P and K were not as high as those under the NPK treatment, recycled manure significantly increased the soil SOM and TN contents and elevated the levels of SMBN (Table 2), but decreased the soil NH_4_^+^-N content, similar to the findings in many previous investigations (Xu et al., 2012; Ma et al., 2020). The main reason for the accumulation of SOM contents under the M and MNPK treatments was that the manure contained a large proportion of recalcitrant organic carbon, which could facilitate the formation of macroaggregates and protect the organic matter from being decomposed (Tao et al., 2017; Li et al., 2020; Lin et al., 2022). High SOM contents provided plentiful nutrients to increase the total abundance of soil microorganisms, which indirectly caused the increase in comammox *Nitrospira* (Wang et al., 2019). Additionally, the application of manure provided available carbon as well as rich and stable nutrients for nitrifier growth, which stimulated comammox *Nitrospira* clade A abundance and promoted the nitrification process, leading to a considerable decrease in the NH_4_^+^-N contents (Han et al., 2018; Li et al., 2020; Lin et al., 2022). Based on comparative genome analysis, some functional genes associated with gluconeogenesis, glycolysis reactions, oxidative TCA cycle and the pentose phosphate pathway were discovered in the core genome of certain comammox *Nitrospira* (Lücker et al., 2010). The *phaZ* gene that encodes polyhydroxybutyrate (PHB) depolymerase was exclusively discovered in comammox *Nitrospira* genomes (Palomo et al., 2018). Overall, this evidence suggests that comammox *Nitrospira* has the potential to grow mixotrophically similar to the other *Nitrospira* (Daims et al., 2001; Koch et al., 2019), so it was not surprising that comammox *Nitrospira* could be shaped by organic manure application. Furthermore, the relative abundances of comammox *Nitrospira* clade A and clade B responded differently to the application of manure, implying that SOM content is a crucial property shaping the niche differentiation of comammox *Nitrospira* in agricultural soil. The application of manure might provide an unfavorable environment for comammox *Nitrospira* clade B grow (He et al., 2022). Notably, other soil properties, such as the available phosphorus content (Liu et al., 2019; Lin et al., 2022), have been found to be vital factors determining the comammox *Nitrospira* community (Figure 3; Supplementary Figure S1). Comammox *Nitrospira* might be more competitive than canonical ammonia oxidizers under phosphorus limited conditions due to possessing the alkaline phosphatase enzyme (Palomo et al., 2016). However, because no isolated comammox strain was obtained from the soil, more efforts determine whether comammox *Nitrospira* can utilize organic substrates directly for growth or which genomic characteristics determine the niche specialization of comammox *Nitrospira* in soil are needed in the future studies.

## 5. Conclusion

Our results showed that diverse long-term fertilization regimes changed the soil properties and resulted in changes in AOB, AOA, *Nitrospira*-like NOB, *Nitrobacter*-like NOB, and comammox *Nitrospira* abundances in Alfisols. Niche separation existed among canonical nitrifying microorganisms and comammox *Nitrospira*. AOB abundances were significantly stimulated by the nitrogen input rates, while the abundances of AOA were increased by recycled manure application. Comammox *Nitrospira* clade A and clade B responded differently to chemical and/or manure fertilizer, and the relative importance of clade B to soil PAO was confirmed by SEM analysis. Variations in the relative abundances of comammox *Nitrospira* clade A and clade B in different soil layers showed that niche differentiation certainly existed between them. Soil pH, NH_4_^+^-N, and SOM contents were crucial factors driving the comammox *Nitrospira* community in Alfisols under long-term fertilization regimes. However, an extension of our knowledge about the contribution to nitrification and the physiological and genomic characteristics of new comammox *Nitrospira* bacteria is needed, and studies of pure comammox *Nitrospira* cultures should be integrated to comprehensively understand the nitrogen cycle under agricultural management.

## Data availability statement

The datasets presented in this study can be found in online repositories. The names of the repository/repositories and accession number(s) can be found at: https://www.ncbi.nlm.nih.gov/, SRR13319769 to SRR13319798.

## Author contributions

YW, XZ, QM, WY, and ZZ performed the field experiment and analyzed the abundances of canonical nitrifiers and comammox *Nitrospira* in soils. YZ, NZ, and LX analyzed and interpreted the effects of different fertilization regimes on soil properties and comammox *Nitrospira* community. YW was the major contributor in writing the manuscript. XZ reviewed and edited the manuscript. All authors read and approved the final manuscript.

## Funding

This work was supported by the National Natural Science Foundation of China (Nos. 41877061, 41907088, and U19A2048), Central Public-Interest Scientific Institution Basal Research Fund (No. BSRF202101), National Key Research and Development Program of China (No. 2021YFD190120303), and the Agricultural Science and Technology Innovation Program (No. CAAS-ASTIP-2016-IEDA).

## Conflict of interest

The authors declare that the research was conducted in the absence of any commercial or financial relationships that could be construed as a potential conflict of interest.

## Publisher’s note

All claims expressed in this article are solely those of the authors and do not necessarily represent those of their affiliated organizations, or those of the publisher, the editors and the reviewers. Any product that may be evaluated in this article, or claim that may be made by its manufacturer, is not guaranteed or endorsed by the publisher.

## Supplementary material

The Supplementary material for this article can be found online at: https://www.frontiersin.org/articles/10.3389/fmicb.2023.1095937/full#supplementary-material
