# Supplementary figures and images for: Differential responses of canonical nitrifiers and comammox Nitrospira to long-term fertilization in an Alfisol of Northeast China
Source: Front Microbiol. 2023 Feb 1;14:1095937. doi: 10.3389/fmicb.2023.1095937 (PMC9929954; doi:10.3389/fmicb.2023.1095937)

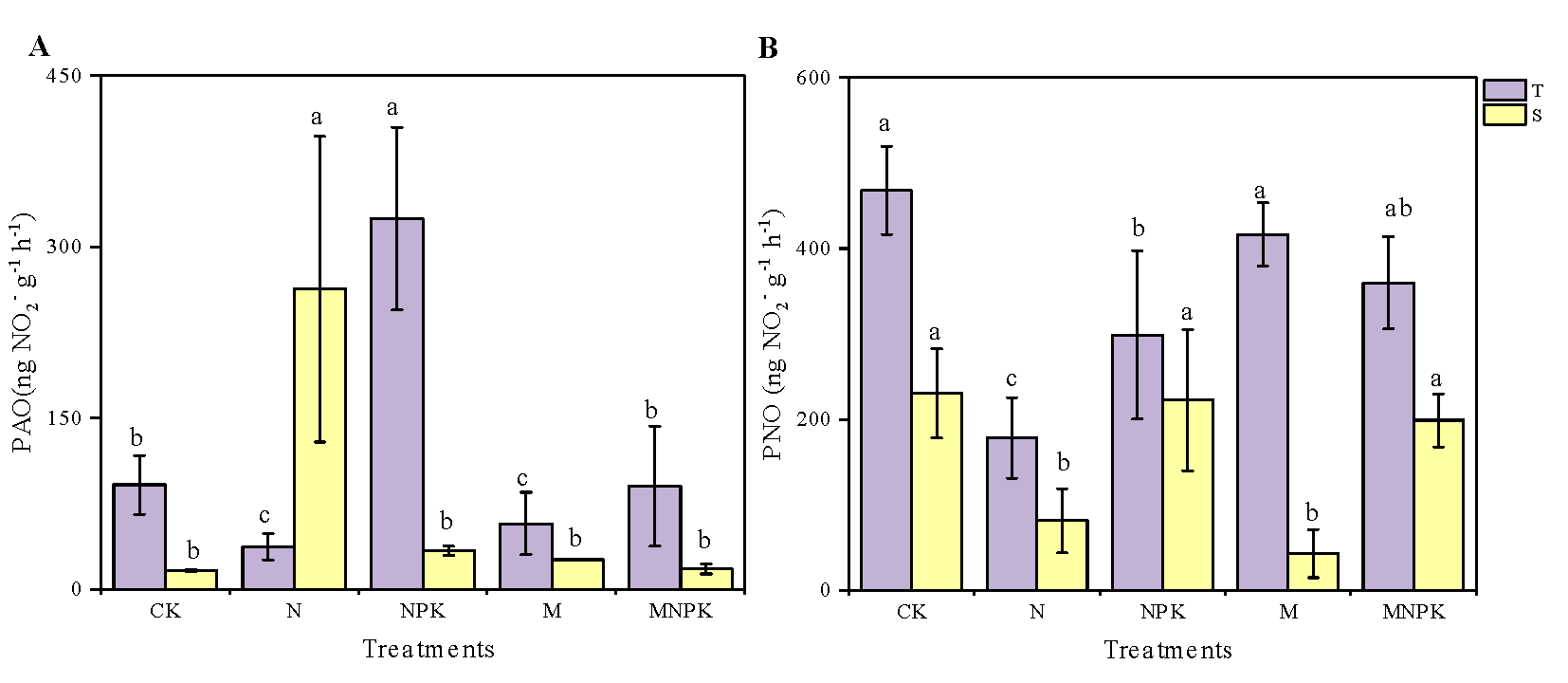

Supplement: Supplementary file 1 [file Presentation_1.ZIP › Figure 1.tif]

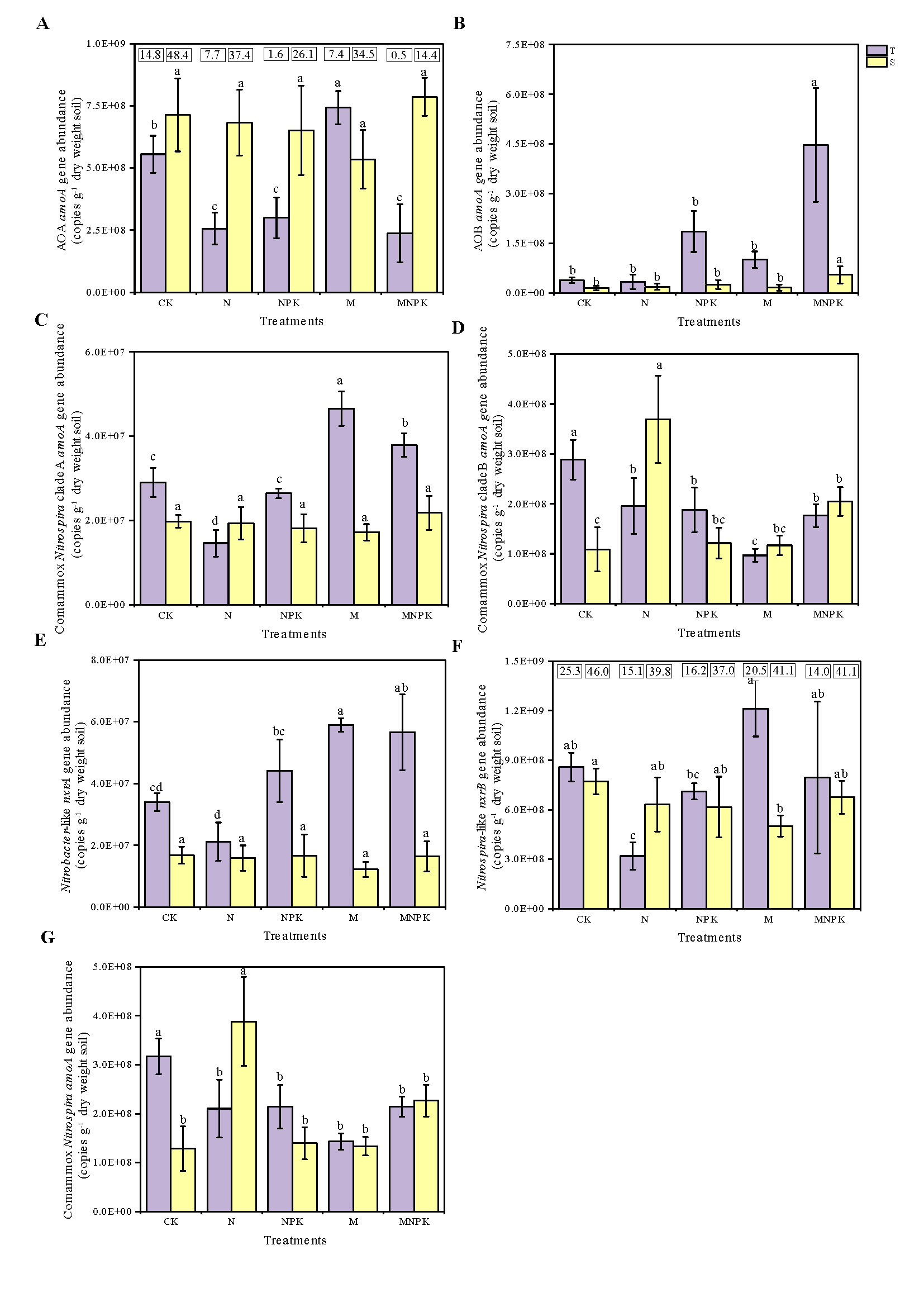

Supplement: Supplementary file 1 [file Presentation_1.ZIP › Figure 2-2023.1.4.tiff]

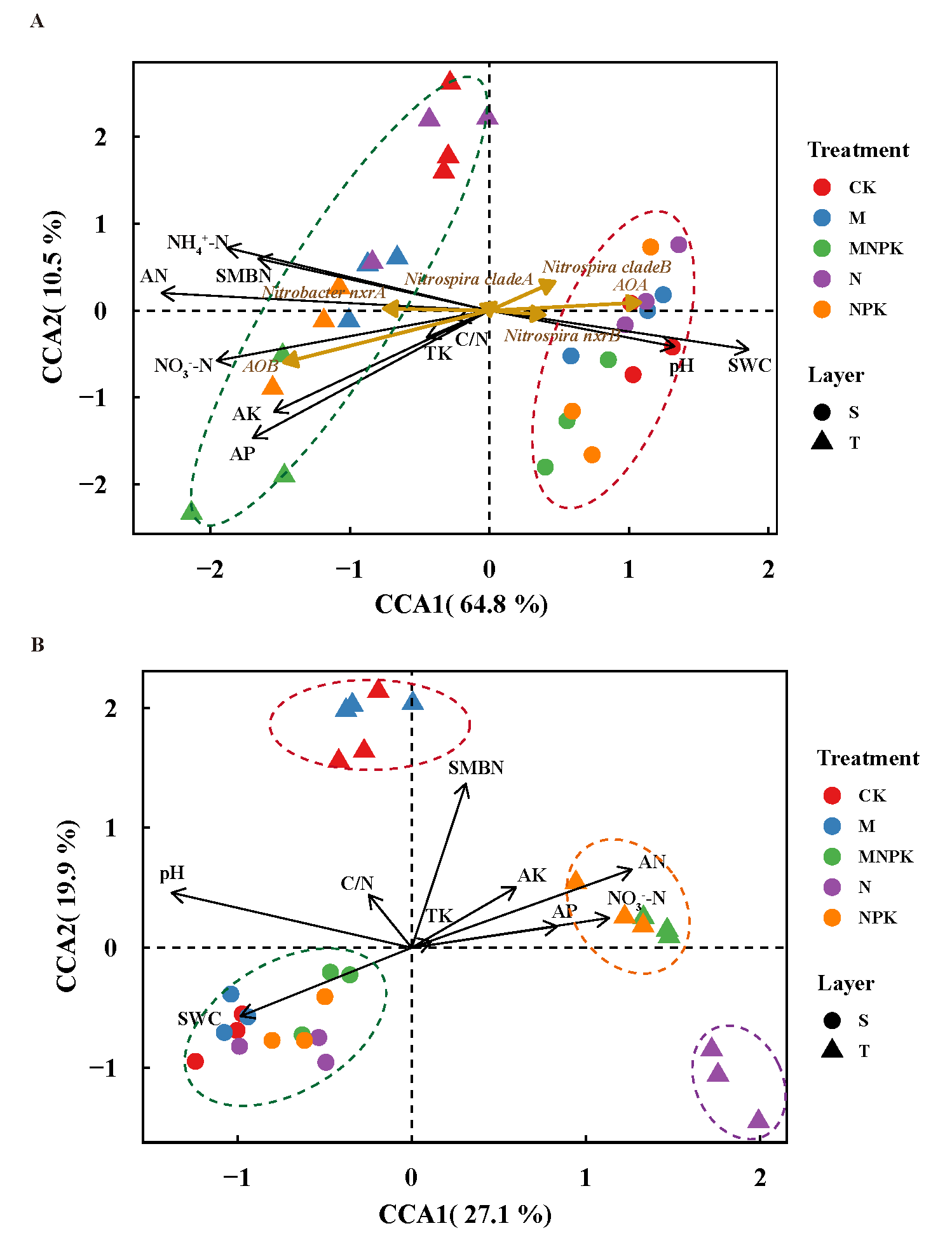

Supplement: Supplementary file 1 [file Presentation_1.ZIP › Figure 3-2023.1.4.tif]

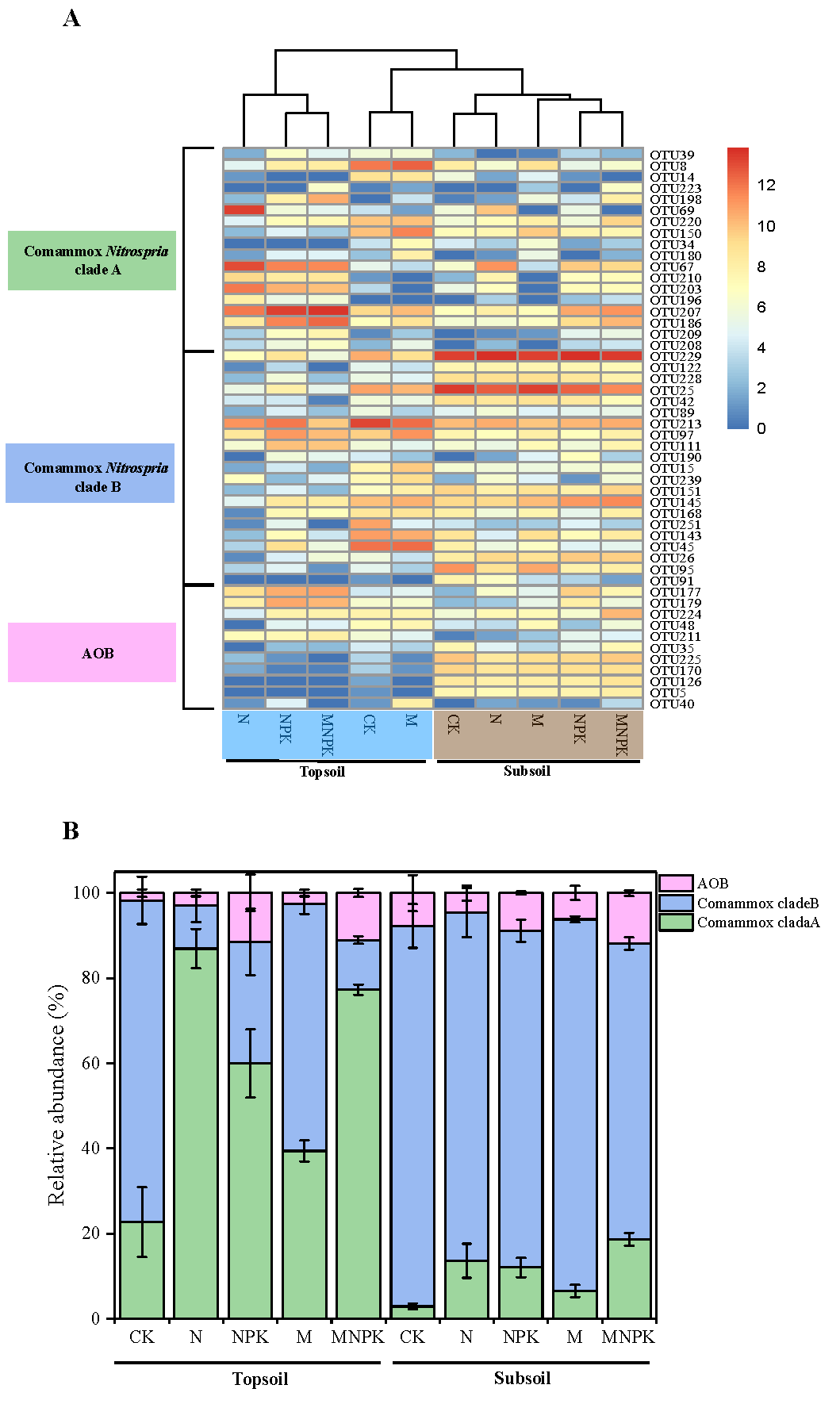

Supplement: Supplementary file 1 [file Presentation_1.ZIP › Figure 4.tiff]

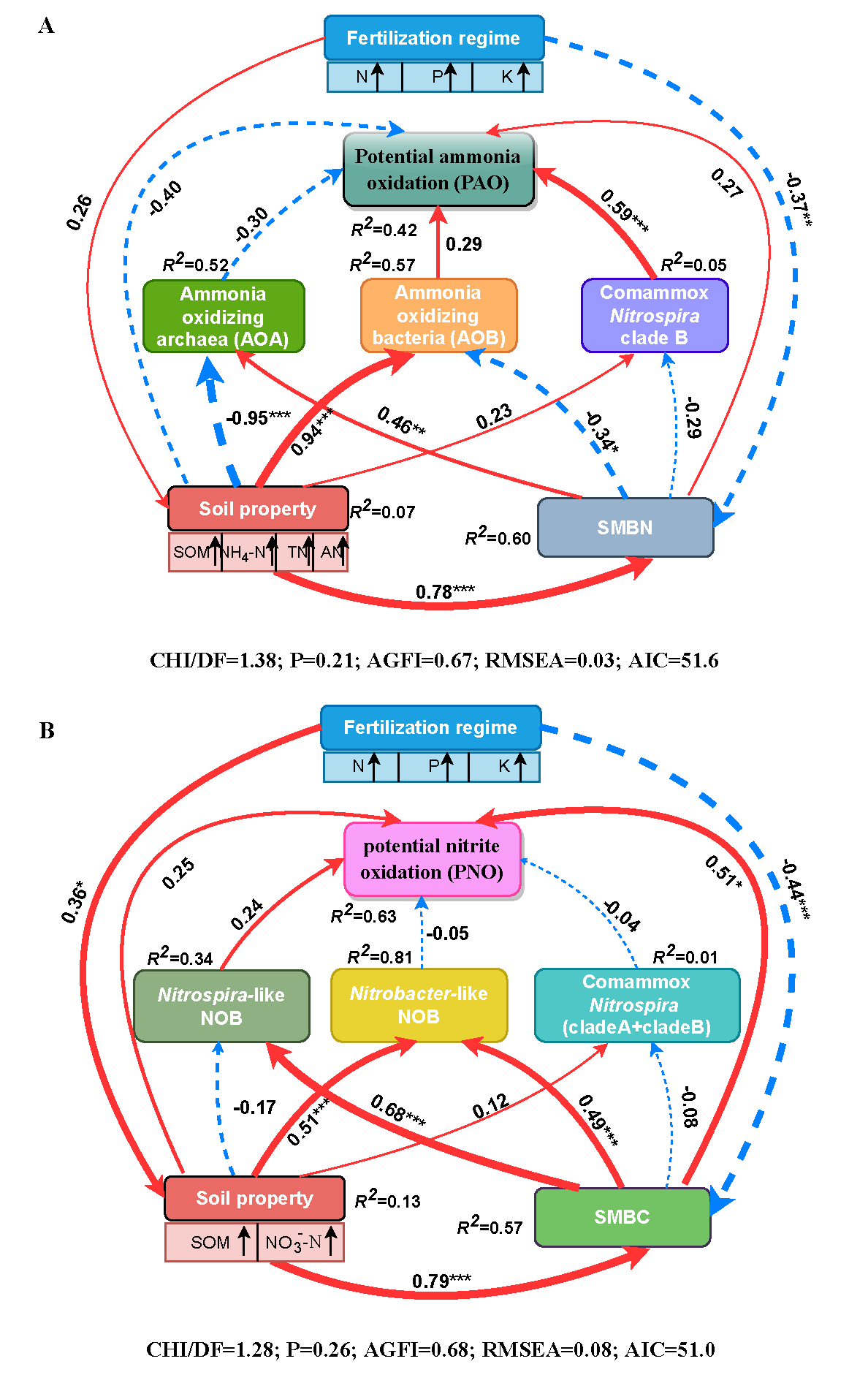

Supplement: Supplementary file 1 [file Presentation_1.ZIP › Figure 5-2023.1.4.tif]

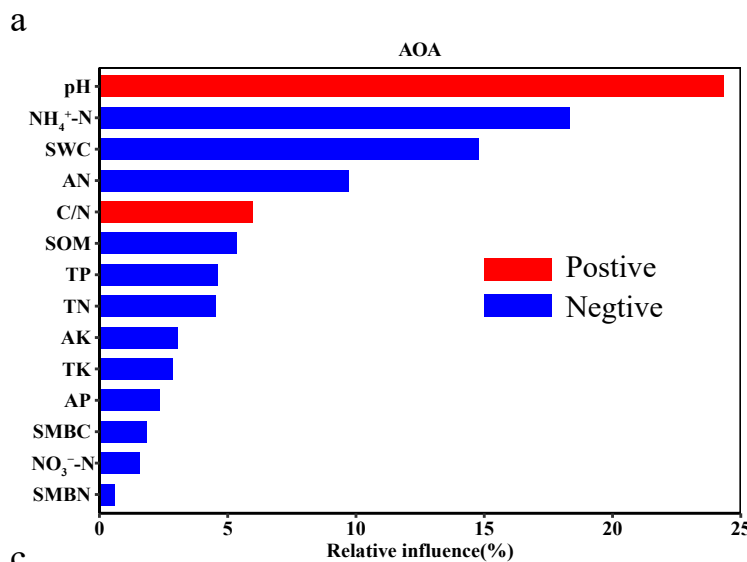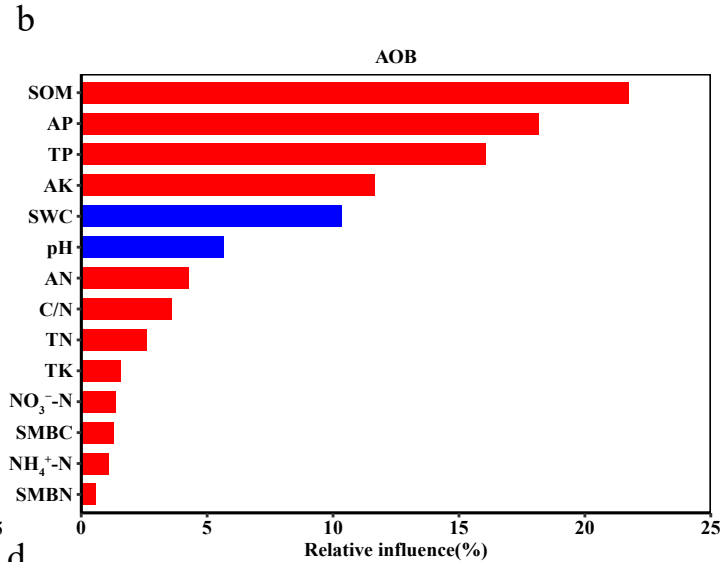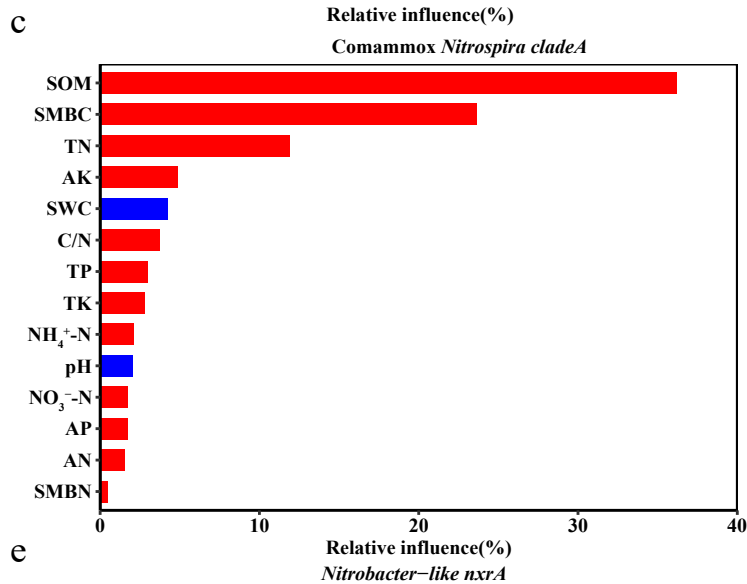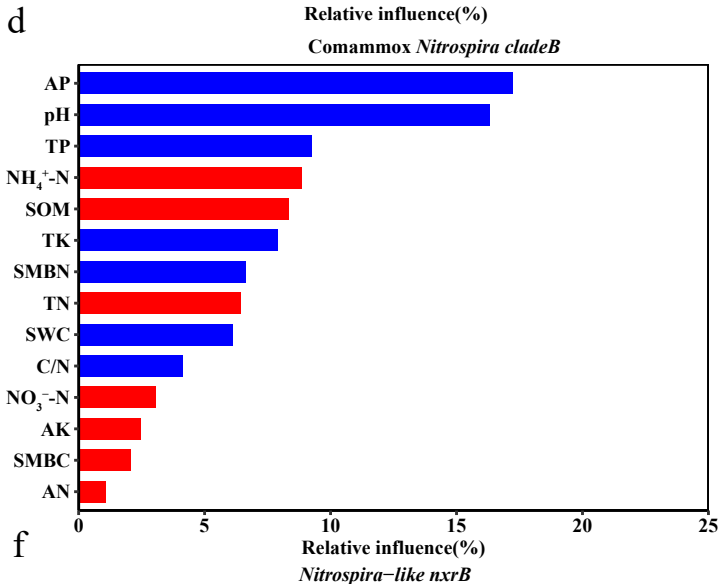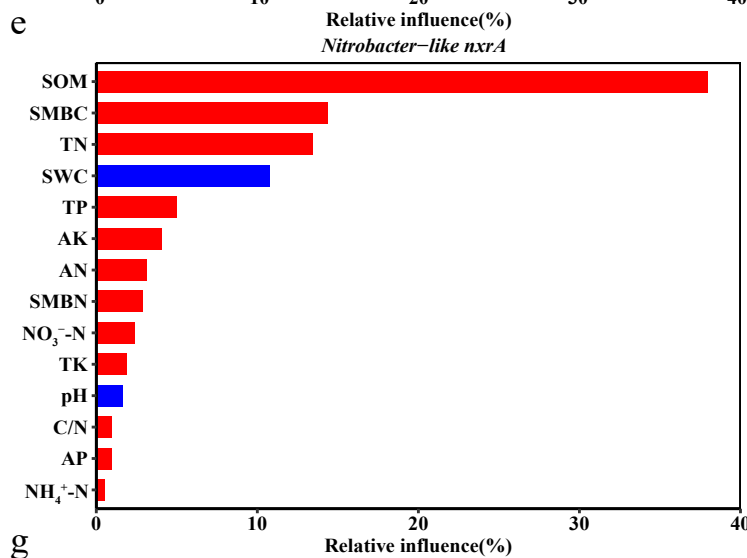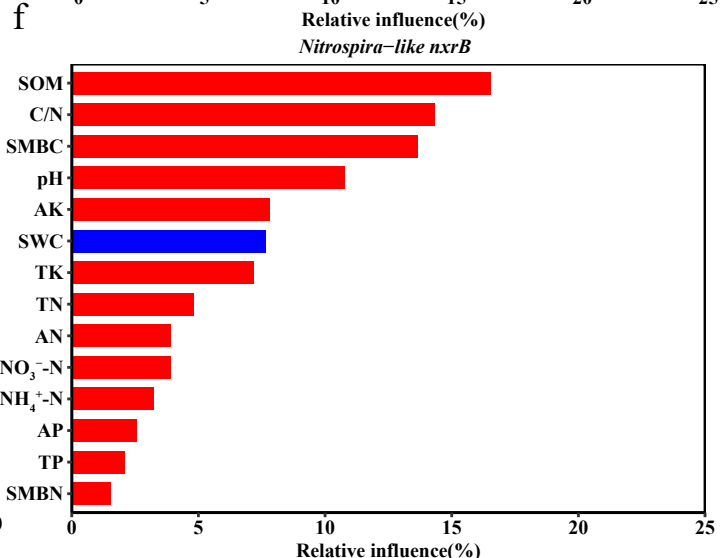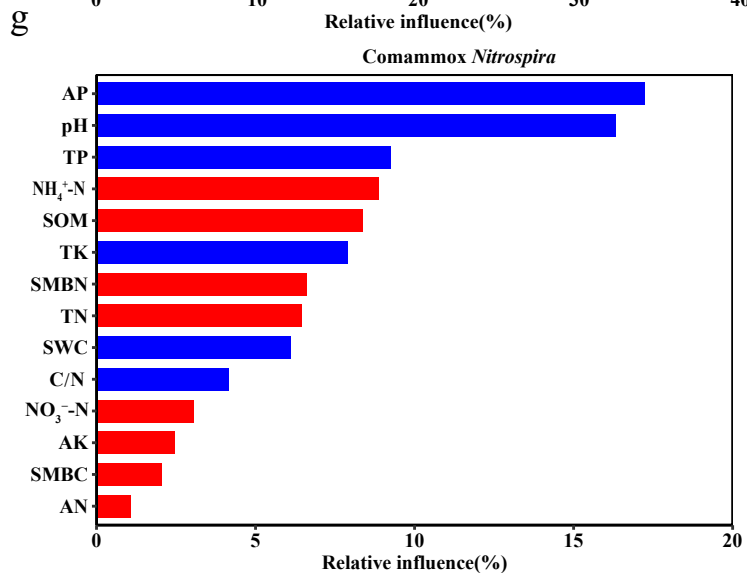

Supplement: Supplementary file 3 [file Image_1.PDF]

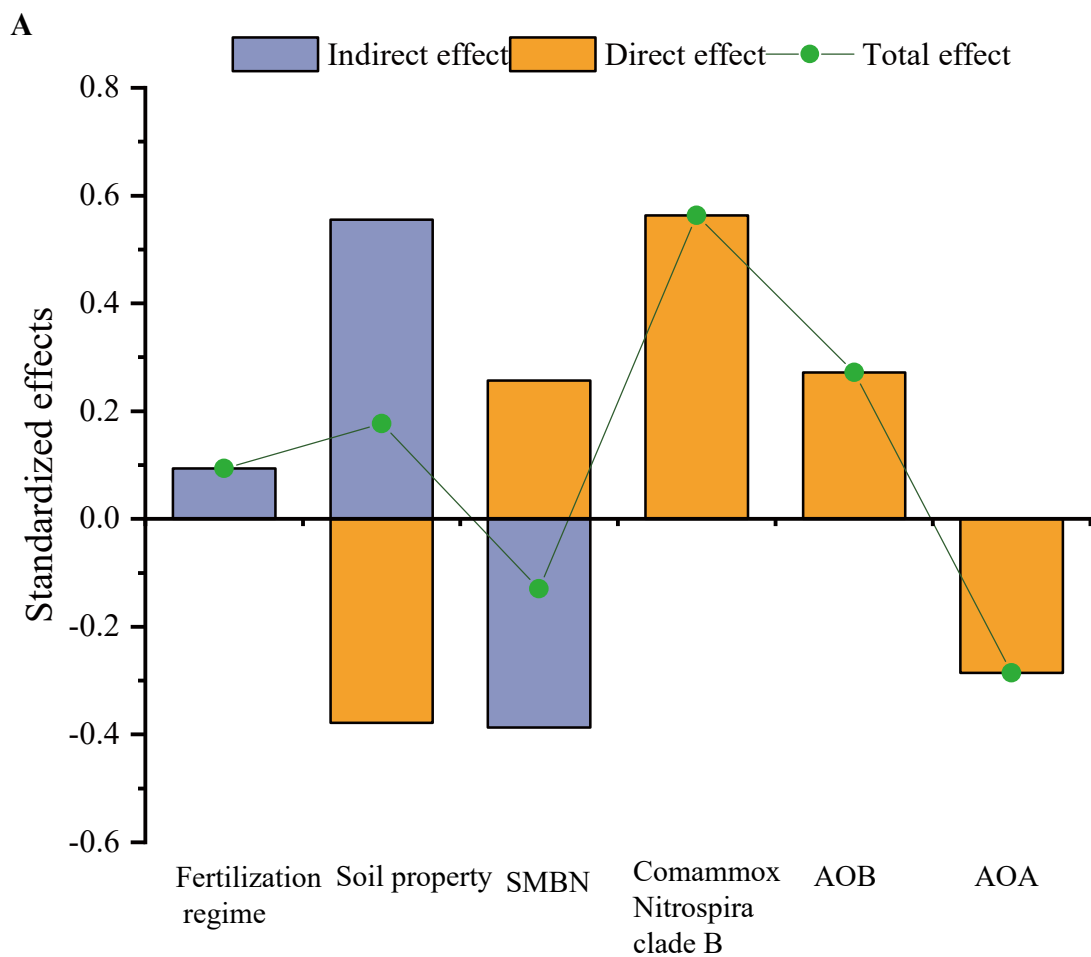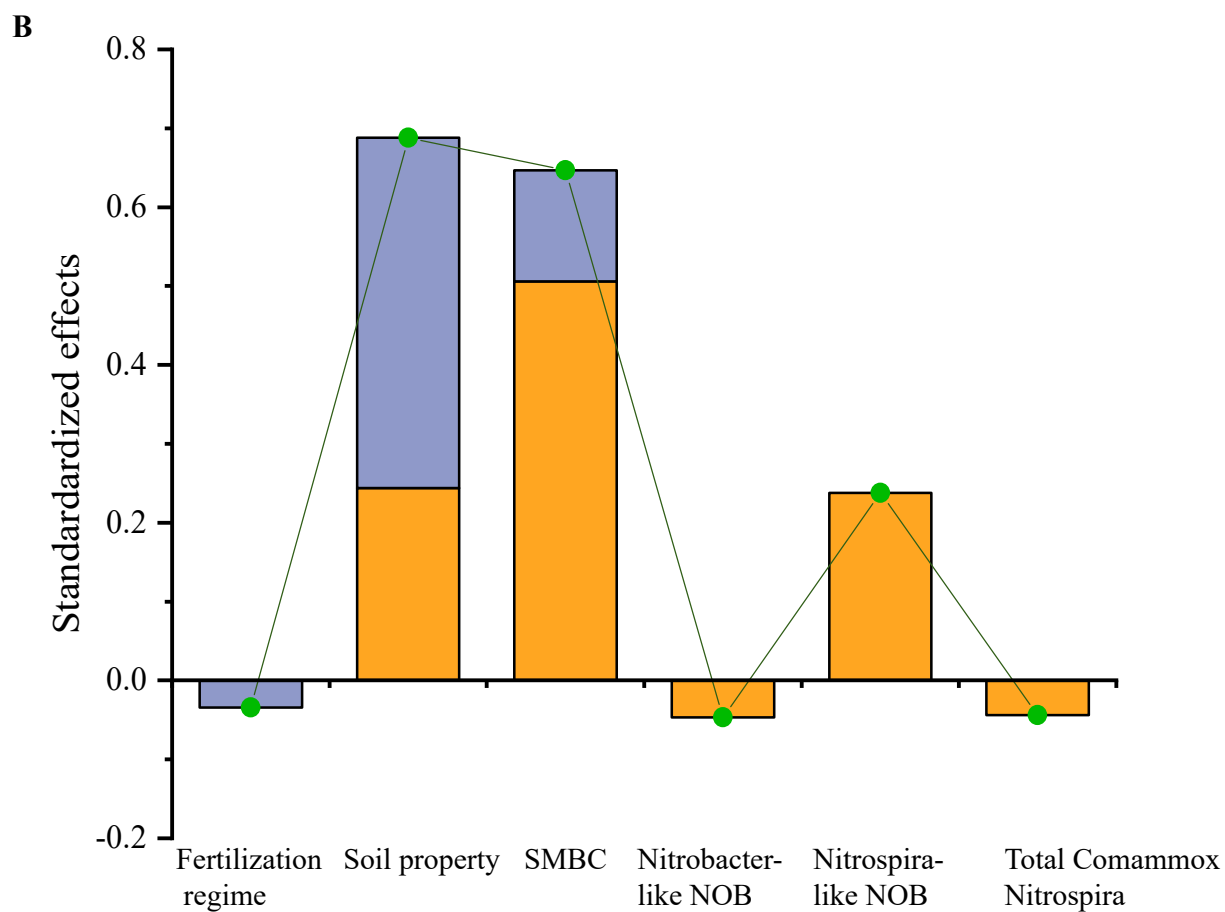

Supplement: Supplementary file 4 [file Image_2.PDF]

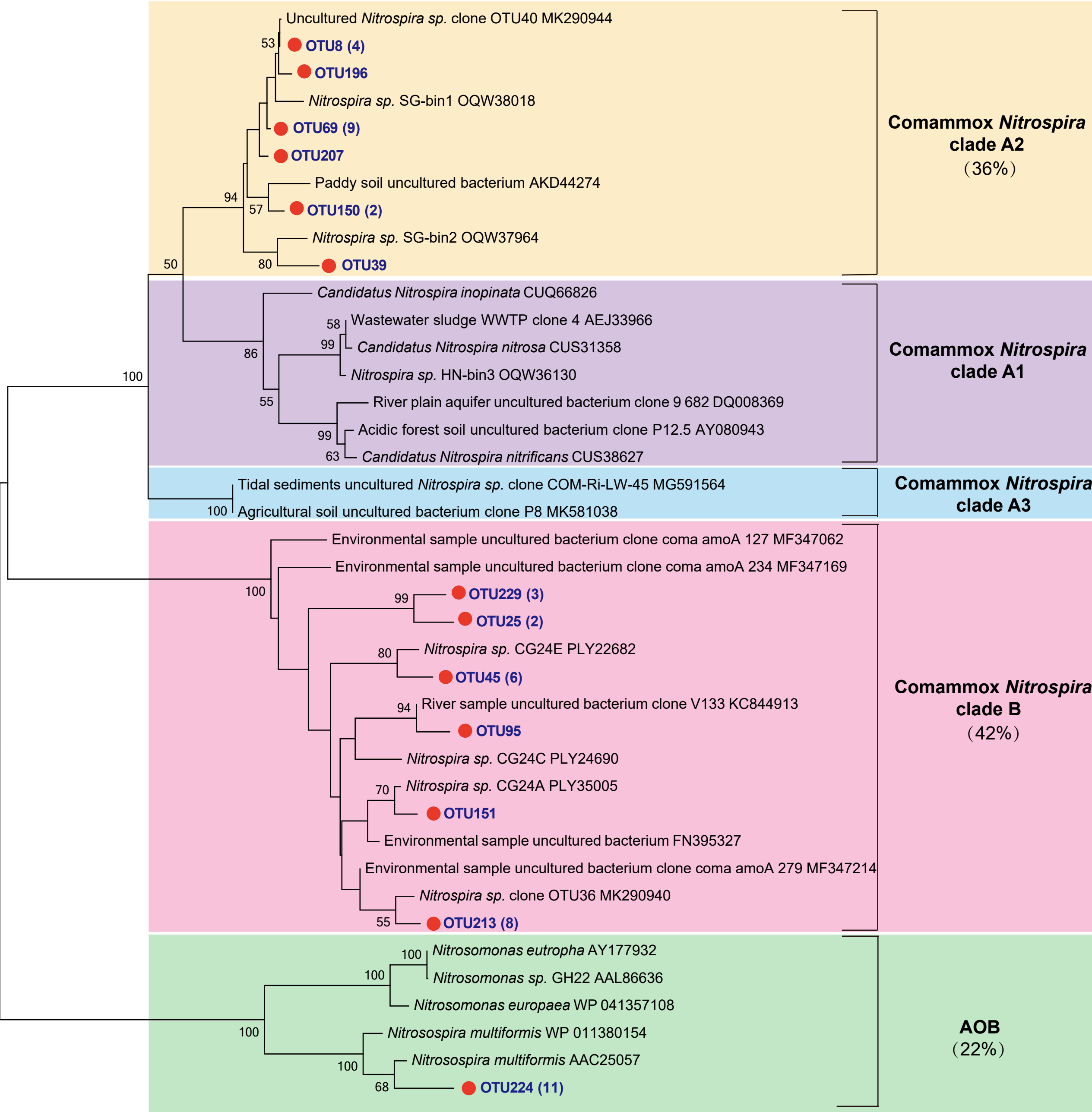

0.050

Supplement: Supplementary file 5 [file Image_3.PDF]

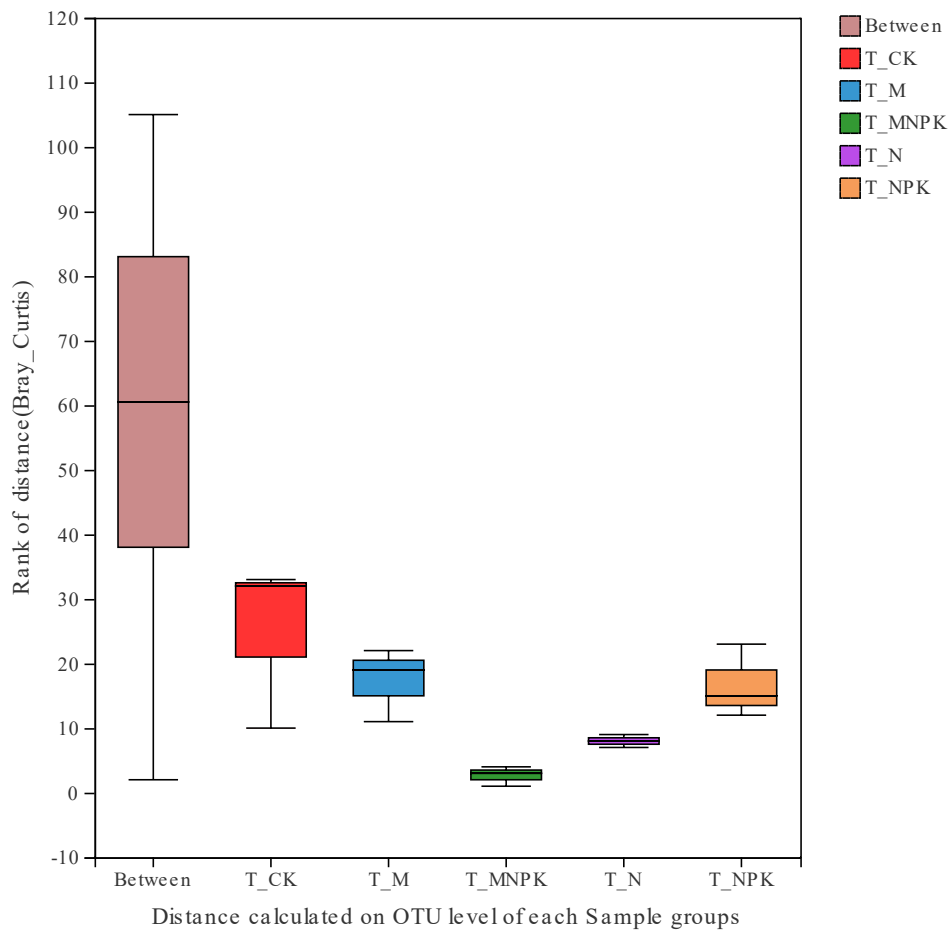

Supplement: Supplementary file 6 [file Image_4.PDF]
