# Supplementary material for: Differential responses of canonical nitrifiers and comammox Nitrospira to long-term fertilization in an Alfisol of Northeast China
Source: Front Microbiol. 2023 Feb 1;14:1095937. doi: 10.3389/fmicb.2023.1095937 (PMC9929954; doi:10.3389/fmicb.2023.1095937)
Supplement: Supplementary file 2 [file Data_Sheet_1.docx]

**Supplementary material**

**Material and Methods**

**Experiment design**

The long-term fertilization experiment was established in 1990 with randomized complete block design, including 12 treatments with three replicated plots. The area of each field plot was 162 m^2^ (9×18 m). Five treatments were selected in this study, including no fertilizer (CK), chemical N fertilizer (N), chemical N, P and K fertilizers (NPK), recycled organic manure (M) and combined application of chemical N, P, K and recycled manure (MNPK) with three replicates. From 1990 to 2011, a three-course rotation pattern was conducted (1×soybean, 2×maize), and each subplot was cultivated in accordance with a soybean-maize-maize sequence. This long-term experiment was similar in design with the Broadbalk experiment at the Rothamsted experimental station. Maize monoculture has been started in each treatment with three replicates since 2012 (Ma et al. 2020).

The input rates of N, P and K fertilizers for maize were 150 kg·N·ha^-1^, 25 kg·P·ha^-1^ and 60 kg·K·ha^-1^ when urea, triple superphosphate and potassium chloride fertilizers were applied, respectively. All P and K fertilizers were basally applied prior to sowing. Urea (40 kg·N·ha^-1^) was applied prior to sowing, and the others were top dressed at the stem-elongation stage. The organic manure used in this experiment was recycled manure derived from nutrient recycling system under the corresponding treatment, which was the distinctive design of this long-term experiment. Briefly, 80% of the harvested grain (the sum production of the three subplots) under M and MNPK treatments was used to feed two pigs in two pens each year, no other exogenous food was allowed to feed the pigs. Meanwhile, 50% of maize straw and 100% of soybean straw derived from the corresponding treatments were ground and padded into the pigsty (only 50% of the integrated maize straw from the three replicated plots was used after maize monoculture). After the grain was consumed completely, all excretion and litter in each pigsty were collected and composted to produce recycled manure. Then, the recycled manure was evenly applied to the initial corresponding treatment before the following spring plowing. Finally, a field nutrient recycling process in M and MNPK treatment was finished, which was consisted of “fertilization-crop harvest-feeding-composting-returning to the fields” steps. Because the amount of recycled manure depended on the crop production under the corresponding treatment in the last year, the inputs rates of the recycled manure varied in accordance with different treatments and different years. From 1991 to 2018, the average annual application rates of N in M and MNPK treatments were 43.7 and 60.2 kg·N·ha^-1^, respectively.

**References**

Francis, C. A., Roberts, K. J., Beman, J. M., Santoro, A. E., and Oakley, B. B. (2005). Ubiquity and diversity of ammonia-oxidizing archaea in water columns and sediments of the ocean. *Proc. Natl. Acad. Sci.* USA 102, 14683-14688. [doi: 10.1073/pnas.0506625102](https://doi.org/10.1073/pnas.0506625102)

Graham, D. W., Knapp, C. W., Vleck, E. S., Bloor, K., Lane, T. B., and Graham, C. E. (2007). Experimental demonstration of chaotic instability in biological nitrification. *ISEM J.* 1, 385-393. doi: 10.1038/ismej.2007.45

Ma, Q., Li, S. L., Xu, Z. Q., Fan, S. B., Xia, Z. Q., Zhou, C. R., et al. (2020). Changes in supply pathways under different long-term fertilization regimes in Northeast China. *Soil Till. Res.* 201, 104609. [doi: 10.1016/j.still.2020.104609](https://doi.org/10.1016/j.still.2020.104609)

Pester, M., Maixner, F., Berry, D., Rattei, T., Koch, H., Lücker, S. (2014). NxrB encoding the beta subunit of nitrite oxidoreductase as functional and phylogenetic marker for nitrite-oxidizing *Nitrospira*. *Environ. Microbiol. Rep.* 16, 3055-3071. [doi:10.1111/1462-2920.12300](https://doi.org/10.1111/1462-2920.12300)

Pjevac, P., Schauberger, C., Poghosyan, L., Herbold, C. W., Van Kessel, M. A., Daebeler, A., et al. (2017). AmoA-targeted polymerase chain reaction primers for the specific detection and quantification of comammox *Nitrospira* in the environment. *Front. Microbiol.* 8, 1508. [doi: 10.3389/fmicb.2017.01508](https://doi.org/10.3389/fmicb.2017.01508)

Poly, F., Wertz, S., Brothier, E., and Degrange, V. (2008). First exploration of Nitrobacter diversity in soils by a PCR cloning-sequencing approach targeting functional gene *nxrA*. *FEMS Microbiol. Ecol.* 63, 132-140. [doi: 10.1111/j.1574-6941.2007.00404.x](https://doi.org/10.1111/j.1574-6941.2007.00404.x)

Rotthauwe, J. H., Witzel, K. P., and Liesack, W. (1997). The ammonia monooxygenase structural gene *amoA* as a functional marker: molecular fine-scale analysis of natural ammonia-oxidizing populations. *Appl. Environ. Microbiol.* 63, 4704-4712. [doi: 10.1128/aem.63.12.4704-4712.1997](https://doi.org/10.1128/aem.63.12.4704-4712.1997)

Wertz, S., Poly, F., Roux, X. L., and Degrange, V. (2008). Development and application of a PCR-denaturing gradient gel electrophoresis tool to study the diversity of *Nitrobacter*-like *nxrA* sequences in soil. *FEMS Microbiol. Ecol.* 63, 261-267. doi: [10.1111/j.1574-6941.2007.00416.x](https://doi.org/10.1111/j.1574-6941.2007.00416.x)

Xia, F., Wang, J. G., Zhu, T., Zou, B., Rhee, S. K., and Quan, Z. X. (2018). Ubiquity and diversity of complete ammonia oxidizer (Comammox). *Appl. Environ. Microbiol.* 84, e01390-18. [doi: 10.1128/AEM.01390-18](https://doi.org/10.1128/AEM.01390-18)

**Supplementary Figure S1 The relative influence of soil properties on nitrifier gene abundances.** The relative influence of soil properties on the *amoA* gene abundances of AOA (A), AOB (B), comammox *Nitrospira* clade A (C), comammox *Nitrospira* clade B (D), the *nxrA* gene abundances of *Nitrobacter*-like NOB (E), the *nxrB* gene abundances of *Nitrospira* -like NOB (F), and total comammox *Nitrospira* (G) are determined by aggregated boosted tree. The red and blue colors stand for the positive and negative effects based on Pearson correlation test, respectively.

**Supplementary Figure S2 Stacked column chart showing the standardized direct effect, indirect effect and total effect on soil PAO (a) and PNO (b) through the structural equation modeling analysis.**

**Supplementary Figure S3 Phylogenetic tree of the translated amino acid sequences of the comammox *Nitrospira amoA* gene constructed by neighbor joining methods.** Sequences from the soil samples were listed with red circles in front of OTU names and highlighted in blue bold. The numbers in the parentheses following OTU names represents the number of sequences with＞98% sequence identity. Significant bootstrap values (>50) based on the neighbor-joining analysis of 1000 resampled data sets are presented next to each node. The scale bar represented 5% sequence divergence.

**Supplementary Figure S4 Rank of Bray-Curtis distance among soil samples under different fertilization regimes.** The letter “T” in front of each treatment name represents the soil from the 0-20cm soil layer.

**Table S1 The qPCR primer sets and thermal conditions used in the study.**

| **Target gene** | **Assay** | **Primer name** | **Primer sequence (5’-3’)** | **Thermal protocol** | **References** |
| --- | --- | --- | --- | --- | --- |
| Archaeal *aomA* (AOA) | qPCR | Arch-amoAF | STAATGGTCTGGCTTAGACG | 95°C for 5 min, 35 cycles of 95°C for 45 s, 53°C for 50 s and 72°C for 1 min. | Francis et al. 2005 |
|  |  | Arch-amoAR | GCGGCCATCCATCTGTATGT |  |  |
| Bacterial *amoA* (AOB) | qPCR | amoA1F | GGGGTTTCTACTGGTGGT | 94°C for 10 min, 35 cycles of 94°C for 30 s, 57°C for 45 s and 72°C for 45 s | Rotthauwe et al. 1997 |
|  |  | amoA2R | CCCCTCKGSAAAGCCTTCTTC |  |  |
| comammox *Nitrospira* clade A *amoA* | qPCR | comaA-244f_a | TACAACTGGGTGAACTA | 95°C for 10 min, 40 cycles of 94°C for 30 s, 58°C for 45 s and 72°C for 1 min | Pjevac et al. 2017 |
|  |  | comaA-244f_b | TATAACTGGGTGAACTA |  |  |
|  |  | comaA-244f_c | TACAATTGGGTGAACTA |  |  |
|  |  | comaA-244f_d | TACAACTGGGTCAACTA |  |  |
|  |  | comaA-244f_e | TACAACTGGGTCAATTA |  |  |
|  |  | comaA-244f_f | TATAACTGGGTCAATTA |  |  |
|  |  | comaA-659r_a | AGATCATGGTGCTATG |  |  |
|  |  | comaA-659r_b | AAATCATGGTGCTATG |  |  |
|  |  | comaA-659r_c | AGATCATGGTGCTGTG |  |  |
|  |  | comaA-659r_d | AAATCATGGTGCTGTG |  |  |
|  |  | comaA-659r_e | AGATCATCGTGCTGTG |  |  |
|  |  | comaA-659r_f | AAATCATCGTGCTGTG |  |  |
| *comammox Nitrospira clade B amoA* | qPCR | comaB-244f_a | TAYTTCTGGACGTTCTA | 95°C for 10 min, 40 cycles of 94°C for 30 s, 50.8°C for 45 s and 72°C for 1 min | Pjevac et al. 2017 |
|  |  | comaB-244f_b | TAYTTCTGGACATTCTA |  |  |
|  |  | comaB-244f_c | TACTTCTGGACTTTCTA |  |  |
|  |  | comaB-244f_d | TAYTTCTGGACGTTTTA |  |  |
|  |  | comaB-244f_e | TAYTTCTGGACATTTTA |  |  |
|  |  | comaB-244f_f | TACTTCTGGACCTTCTA |  |  |
|  |  | comaB-659r_a | ARATCCAGACGGTGTG |  |  |
|  |  | comaB-659r_b | ARATCCAAACGGTGTG |  |  |
|  |  | comaB-659r_c | ARATCCAGACAGTGTG |  |  |
|  |  | comaB-659r_d | ARATCCAAACAGTGTG |  |  |
|  |  | comaB-659r_e | AGATCCAGACTGTGTG |  |  |
|  |  | comaB-659r_f | AGATCCAAACAGTGTG |  |  |
| *Nitrobacter-*like *nxrA* | qPCR | F1norA | CAGACCGACGTGTGCGAAAG | 94°C for 5 min, 40 cycles of 94°C for 30 s, 52°C for 45 s and 72°C for 1min | Poly et al. 2008 |
|  |  | R2norA | TCCACAAGGAACGGAAGGTC |  | Wertz et al. 2008 |
| *Nitrospira-*like *nxrB* | qPCR | nxrB169F | TACATGTGGTGGAACA | 94°C for 5 min, 40 cycles of 94°C for 30 s, 56.2°C for 45 s and 72°C for 45 s | Pester et al. 2014 |
|  |  | nxrB638R | CGGTTCTGGTCRATCA |  |  |
| *comammox Nitrospira amoA* | High throughput sequencing (partial nested PCR) | ComaA189Y | GGNGACTGGGAYTTYTGG | 94°C for 5 min, 40 cycles of 94°C for 1 min, 52°C for 50 s and 72°C for 50 s | Xia et al. 2018 |
|  |  | ComaC576R | GAAGCCCATRTARTCNGCC |  |  |
|  |  | ComaA209F | GAYTGGAARGAYCGNCA | 94°C for 5 min, 40 cycles of 94°C for 1 min, 52°C for 50 s and 72°C for 50 s |  |
|  |  | ComaC576R | GAAGCCCATRTARTCNGCC |  |  |

**Table S2 The effects of soil layer and fertilization regime on potential nitrification rates, gene abundances were analyzed by two-way ANOVA and least significant difference test at a 0.95 confidence level.**

|  |  | **AOA** | **AOB** | **comammox *Nitrospira* cladeA** | **comammox *Nitrospira* cladeB** | **comammox *Nitrospira*** | ***Nitrobacter-nxrA*** | ***Nitrospira-nxrB*** | **PAO** | **PNO** |
| --- | --- | --- | --- | --- | --- | --- | --- | --- | --- | --- |
| **Soil layer**  **(T1)** | *F* | 39.05 | 38.16 | 105.37 | 0.09 | 0.98 | 138.51 | 4.36 | 5.90 | 84.72 |
|  | *P* | **＜0.001** | **＜0.001** | **＜0.001** | 0.77 | 0.34 | **＜0.001** | **0.05** | **0.025** | **＜0.001** |
| **Fertilization (T2)** | *F* | 3.40 | 14.74 | 22.22 | 13.13 | 10.67 | 8.31 | 4.02 | 8.03 | 12.16 |
|  | *P* | **0.028** | **＜0.001** | **＜0.001** | **＜0.001** | **＜0.001** | **＜0.001** | **0.015** | **＜0.001** | **＜0.001** |
| **T1×T2** | *F* | 10.43 | 10.07 | 24.10 | 13.26 | 13.26 | 10.46 | 5.95 | 16.70 | 6.92 |
|  | *P* | **＜0.001** | **＜0.001** | **＜0.001** | **＜0.001** | **＜0.001** | **＜0.001** | **0.003** | **＜0.001** | **＜0.001** |

AOA: ammonia‐oxidizing archaea; AOB: ammonia‐oxidizing bacteria; comammox *Nitrospira*: the sum of *amoA* gene abundances of comammox *Nitrospira* clade A and clade B; PAO: potential ammonia oxidation; PNO: potential nitrite oxidation.

**Table S3 Spearman correlation between soil potential ammonia oxidation (PAO), potential nitrite oxidation (PNO) and nitrifier gene abundances.**

| **Spearman correlation** | **Spearman’s**  **r and P value** | **AOA** | **AOB** | **comammox *Nitrospira* cladeA** | **comammox *Nitrospira* cladeB** | **comammox *Nitrospira*** | ***Nitrobacter*-*nxrA*** | ***Nitrospira*-*nxrB*** |
| --- | --- | --- | --- | --- | --- | --- | --- | --- |
| PAO | *r* | **-0.373*** | **0.392*** | 0.351 | **0.394*** | **0.479**** | **0.469**** | 0.125 |
|  | *P* | 0.042 | 0.032 | 0.057 | 0.031 | 0.007 | 0.009 | 0.511 |
| PNO | *r* | -0.099 | **0.590**** | **0.730**** | -0.031 | 0.074 | **0.717**** | **0.595**** |
|  | *P* | 0.604 | 0.001 | 0.001 | 0.869 | 0.699 | 0.001 | 0.001 |

Bold values indicate significant correlations (*P*＜0.05). * and ** represent the significance at 0.05, 0.01 level, respectively.

**Table S4 The theoretical ammonia concentrations in soil aqueous solution.**

| **Soil Layer (cm)** | **Treatments** | **Soil pH** | **Soil water content (SWC)** | **NH_4_^+^-N**  **(mg·kg^-1^ soil)** | **Theoretic ammonia concentration (nM)** |
| --- | --- | --- | --- | --- | --- |
| 0-20 | CK | 6.92 (0.05)a | 17.55 (0.96)a | 9.76 (0.03)d | 14270 |
|  | N | 5.16 (0.21)d | 16.84 (4.02)a | 13.56 (0.35)a | 400 |
|  | NPK | 6.24 (0.05)b | 17.45 (1.72)a | 10.72 (0.57)c | 3353 |
|  | M | 6.90 (0.08)a | 18.24 (0.96)a | 7.99 (0.33)e | 9835 |
|  | MNPK | 5.87 (0.19)c | 17.58 (0.96)a | 11.74 (1.00)b | 1669 |
| 20-40 | CK | 7.02 (0.14)a | 21.25 (1.26)a | 5.94 (0.38)c | 9512 |
|  | N | 6.50 (0.21)b | 20.78 (1.96)a | 7.17 (0.06)a | 3560 |
|  | NPK | 6.89 (0.12)a | 21.13 (1.56)a | 6.55 (0.15)b | 7838 |
|  | M | 7.01 (0.06)a | 20.92 (0.32)a | 5.66 (0.09)c | 8772 |
|  | MNPK | 6.85 (0.13)a | 20.93 (1.45)a | 7.39 (0.31)a | 7845 |

The theoretical ammonia concentrations in soil aqueous solution are calculated according to the ionization equilibrium (NH_4_^+^ ↔ NH_3_ + H^+^; pK_a_ = 9.25 at 25℃)


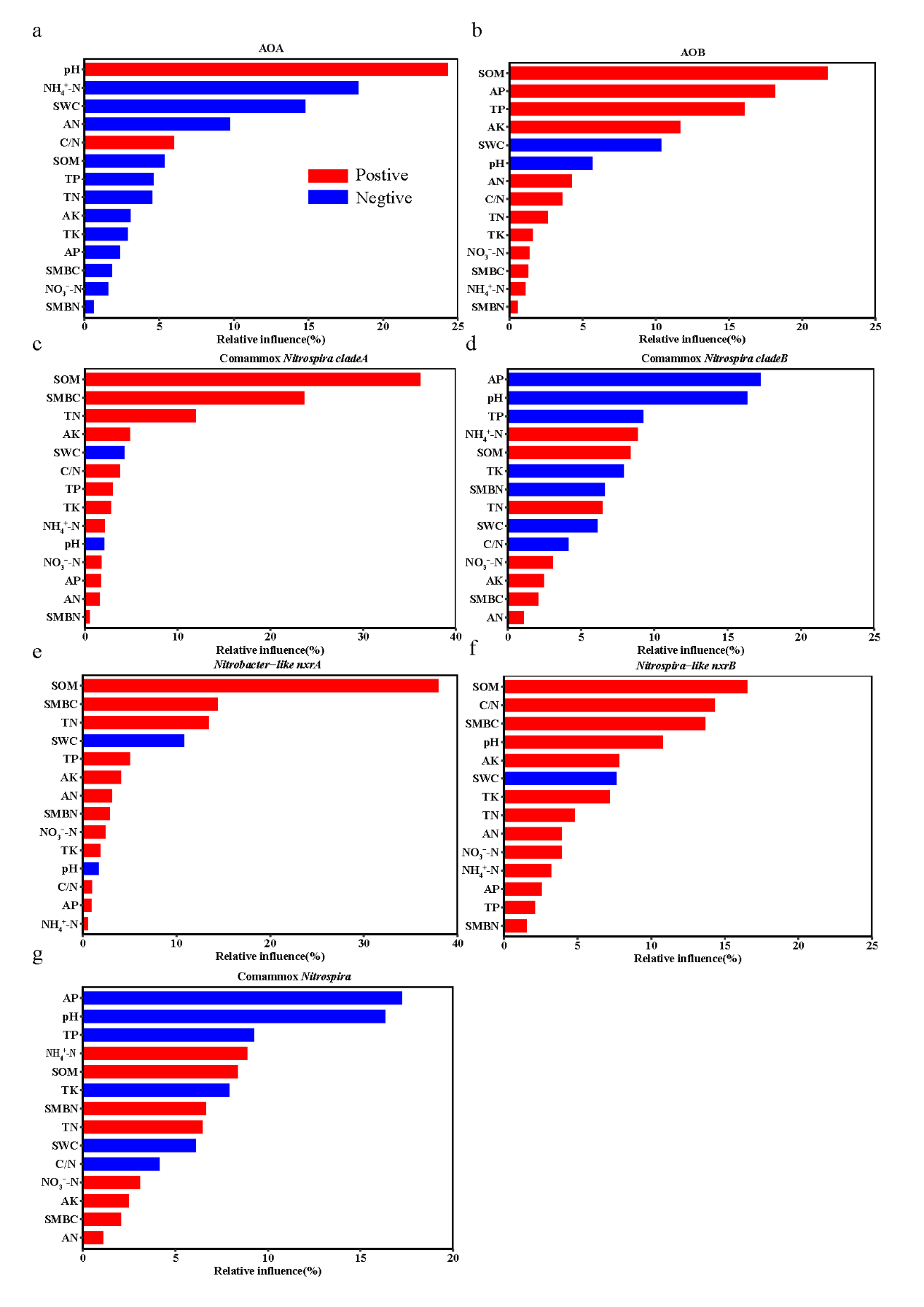


**Supplementary Figure S1**


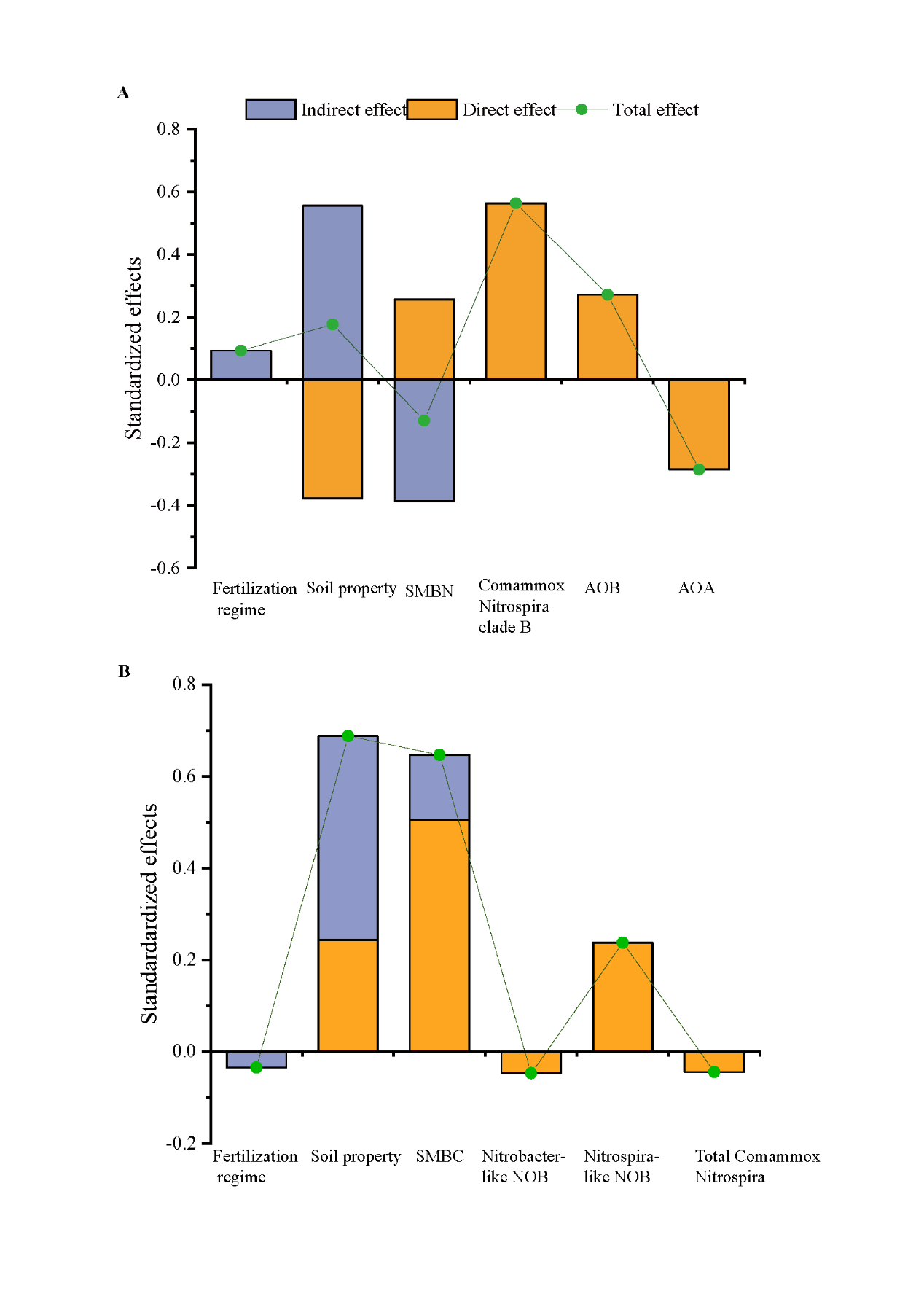


**Supplementary Figure S2**


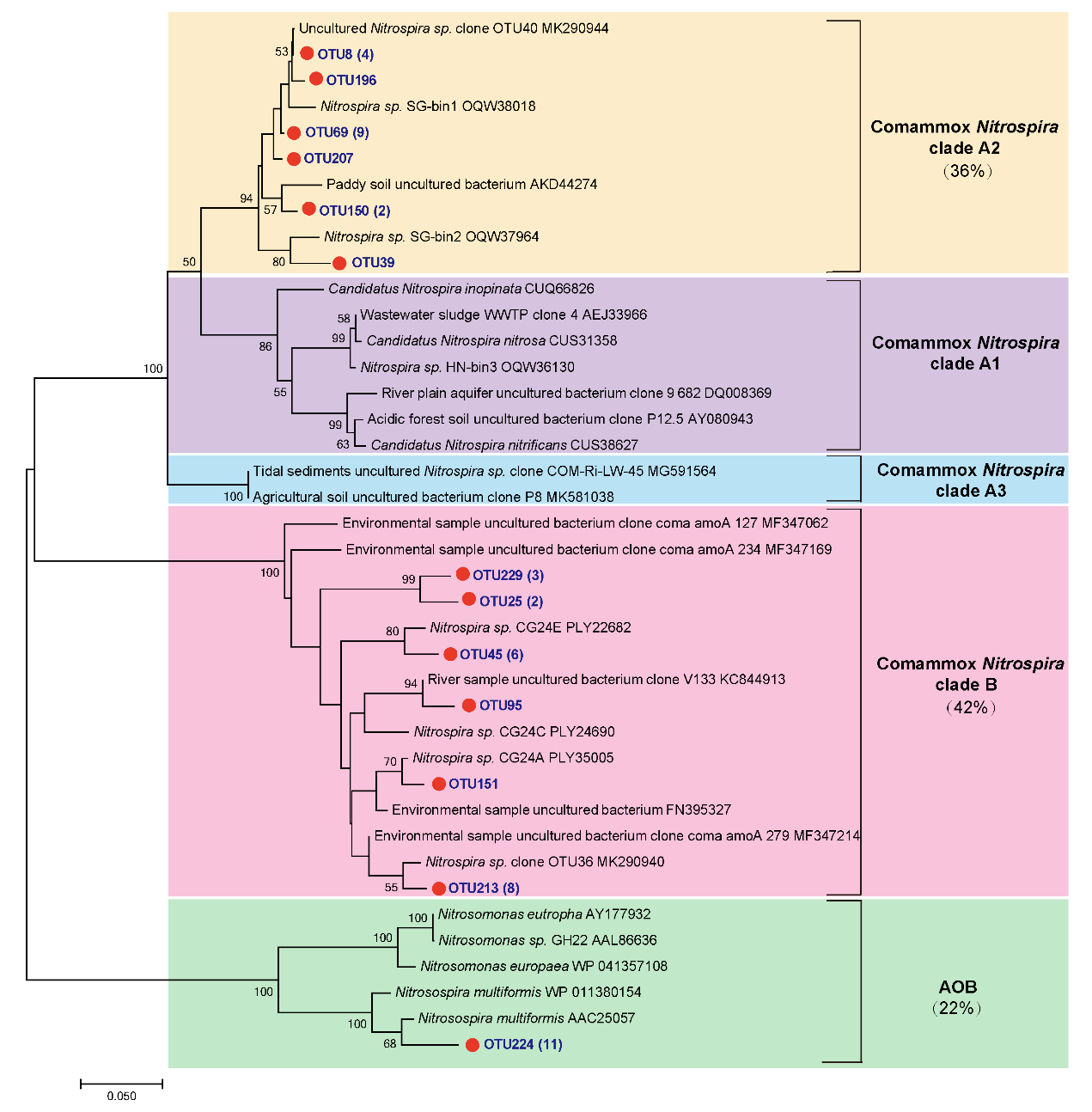


**Supplementary Figure S3**


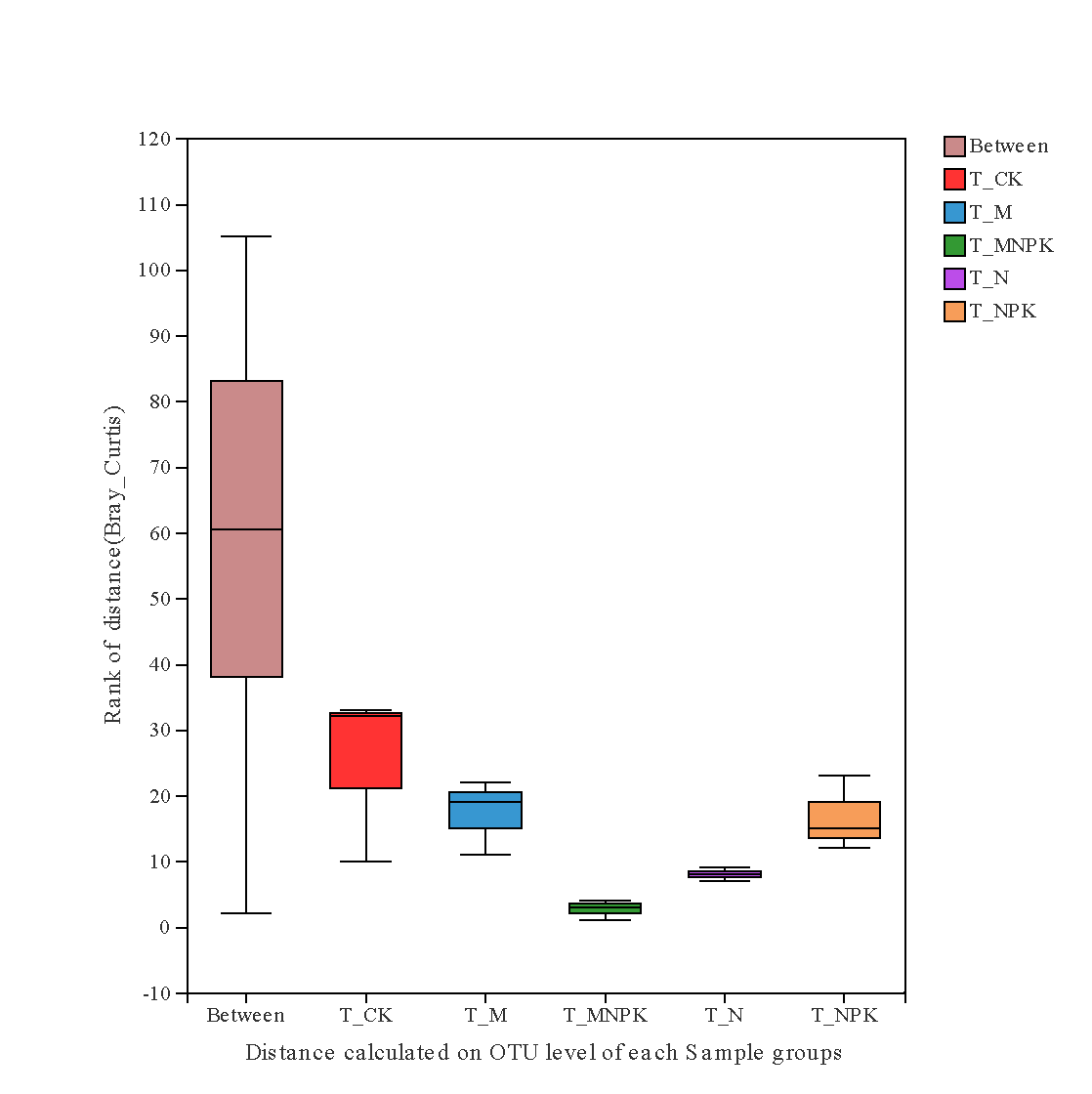


**Supplementary Figure S4**
